# A structure-based modeling approach identifies effective drug combinations for RAS-mutant acute myeloid leukemia

**DOI:** 10.1016/j.isci.2026.116875

**Published:** 2026-07-20

**Authors:** Luke Jones, Oleksii Rukhlenko, Tânia Dias, Hiroaki Imoto, Ciardha Carmody, Kieran Wynne, Boris N. Kholodenko, Jonathan Bond

**Affiliations:** 1 Systems Biology Ireland, Dublin, Ireland; 2 School of Medicine, University College Dublin, Dublin, Ireland; 3 SFI Centre for Research Training in Genomics Data Science, NUI Galway, Galway, Ireland; 4 Department of Pharmacology, Yale University School of Medicine, New Haven, CT, USA; 5 Children’s Health Ireland at Crumlin, Dublin, Ireland

**Keywords:** acute myeloid leukemia, ras, structure-based modeling, drug combination

## Abstract

Mutations activating RAS/RAF/MEK/ERK signaling confer poor outcomes in acute myeloid leukemia (AML), but targeting this pathway is challenging. We used a structure-based, dynamic RAS pathway model to predict RAF inhibitor (RAFi) combinations that synergistically suppress RAS-mutant AML. *In silico* models predicted synergy for two iterations of conformation-specific RAFi’s which were validated *in vitro*. Lifirafenib (type II) + encorafenib (type I½) was highly synergistic against *NRAS*- and *KRAS*-mutant AML cells, while lifirafenib + SB590885 (type I) synergy was *NRAS*-mutant-specific. Combination efficacy correlated with measured RAS pathway activity. Leveraging *in silico* pharmacokinetic predictions, we tested RAFi combinations in an *NRAS*-mutant AML patient-derived xenograft, finding improved leukemia growth delay and survival compared with single agents. Both combinations showed site-specific efficacy against circulating and spleen-resident blasts. In summary, our integrated modeling approach effectively identified non-obvious RAFi combinations that are effective *in vitro* and *in vivo*, thereby suggesting alternative therapeutic strategies for RAS-mutant AML.

## Highlights

• Combined conformation-specific RAF inhibitors suppress RAS signaling in *RAS*-mut AML
• Suppression corresponds to synergistic killing of *RAS*-mutant AML cell lines
• RAF inhibitor combinations show site-specific efficacy in *RAS*-mut AML PDX

## Graphical abstract

**Figure:**
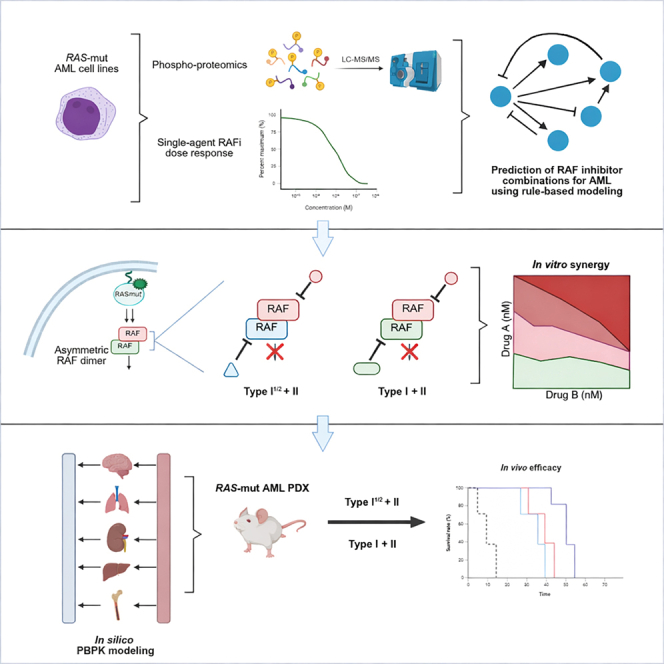

## Teaser

Therapeutics; Structural biology; Cancer.

## Introduction

Mutations that activate RAS signaling are common in myeloid malignancies and are typically associated with a proliferative phenotype and aggressive disease.^1^^,^^2^^,^^3^^,^^4^ These mutations are frequent in acute myeloid leukemia (AML) regardless of age, with evolving evidence suggesting that RAS pathway activation alters therapy responsiveness. In line with this, mutations in *NRAS* and *KRAS*, and in regulators of RAS signaling such as *NF1* and *PTPTN11*, have been associated with poor outcomes.^5^^,^^6^^,^^7^ Additionally, *RAS* mutations have been implicated in the development of resistance to newer targeted therapies, including FLT3 and IDH1/2 inhibitors.^8^^,^^9^^,^^10^

These prognostic associations have driven efforts to address the currently unmet need of targeting aberrant RAS signaling in AML and other cancers. Prior to the recent development of direct inhibitors of RAS, which are largely untested in the context of myeloid malignancies, RAS proteins were considered “undruggable.” Therefore, efforts to inhibit aberrant RAS signaling have focused on targeting factors upstream and downstream of RAS. Of these, MEK inhibition has shown most promise, exhibiting modest efficacy in preclinical models by reducing downstream ERK phosphorylation and proliferation.^11^^,^^12^ Targeting RAF, the kinase immediately downstream of RAS, has been less effective due to the phenomenon of paradoxical activation, whereby single-agent RAF inhibitors cause increased pathway activity and intrinsic or rapidly acquired resistance.^13^^,^^14^^,^^15^

**Figure 1:**
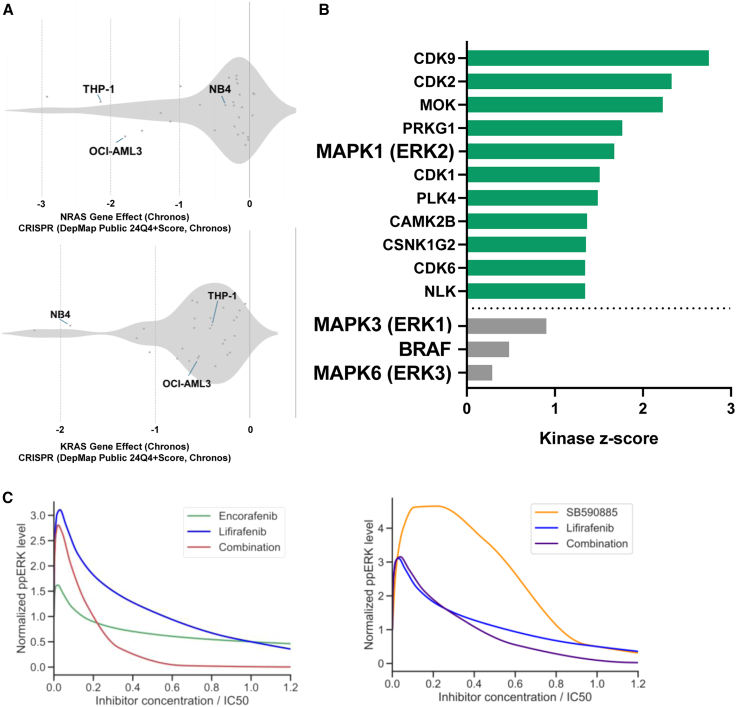
Structure-based modeling predicts RAF inhibitor combinations effective against *RAS*-mutant AML (A) Gene essentiality scores for *NRAS* (top panel) and *KRAS* (bottom panel) across AML cell lines, ranked using data available from CRISPR screens in DepMap (https://depmap.org/portal/). (B) Kinase set enrichment analysis (KSEA) comparing kinase enrichment in *RAS*-mutant (*n* = 5) versus *RAS*-WT (*n* = 3) cell lines. Green bars indicate kinases significantly enriched (*p* < 0.1) in *RAS*-mutant cells. Gray bars represent RAS pathway components enriched in *RAS*-mutant cells that did not meet the statistical threshold (*p* < 0.1). (C) Steady-state phosphorylated ERK (ppERK) responses to RAF inhibitor treatments. Inhibitor concentrations were normalized to their respective IC_50_ values. The equilibrium dissociation constant (Kd) for SB590885 (type I) is reported as 0.3 nM. For encorafenib (type I½) and lifirafenib (type II), Kd values are not publicly available; therefore, we approximated Kd using available EC_50_ values from SelleckChem. The Kd values used in the model were 0.3 nM for SB590885, 4 nM for encorafenib, and 23 nM for lifirafenib.

To overcome this, we recently reported a structure-based *in silico* modeling approach for identifying non-obvious RAF kinase inhibitor combinations that effectively suppress oncogenic RAS signaling while preventing paradoxical pathway activation.^16^ As with other kinases, RAF toggles between inactive and active conformations, which differ by the relative positions of the highly conserved DFG motif and αC helix. ATP-competitive RAF inhibitors are classified based on their preference for binding to alternate (IN or OUT) conformations of the RAF kinase ATP-binding pocket (IN and OUT positions correspond to active and inactive kinase conformations, respectively) (Figure 1).^17^^,^^18^^,^^19^ There are three distinct RAF inhibitor types, specifically targeting: αC-IN/DFG-IN (denoted CI/DI, Type I), αC-OUT/DFG-IN (CO/DI, Type I½), and αC-IN/DFG-OUT (CI/DO, Type II).

Importantly, we and others showed that the paradoxical activation of downstream ERK signaling in response to single-agent RAF inhibitors is primarily due to RAF dimer asymmetry. As each protomer exists in an alternate conformation (IN-OUT), use of a single RAF inhibitor will bind one protomer and cause allosteric activation of the other.^20^^,^^21^^,^^22^^,^^23^ Our structure-based modeling approach showed that RAF dimer asymmetry is a targetable vulnerability, with the combination of structurally different RAF inhibitors abrogating paradoxical activation and significantly reducing downstream pathway activation and cell viability in *RAS*- and *RAF*-mutant solid tumor models.^16^^,^^24^

Given the prevalence and poor prognosis linked to RAS signaling mutations in AML, we now apply our modeling approach to leukemia to address an unmet clinical need. By refining and adapting our structure-based approach by integrating AML-specific kinase pathway activities, we have now identified RAF inhibitor combination approaches that effectively kill *RAS*-mutant AML *in vitro* and in preclinical *in vivo* models.

## Results

### Structure-based modeling identifies dual RAFi combinations for RAS-mutant AML

We first evaluated the contribution of oncogenic *RAS* mutations to the proliferation and survival of leukemia cells. We assessed *RAS* essentiality in AML cell lines using DepMap^25^ (https://depmap.org/portal) to help us select the optimum *in vitro* models. Of AML cell lines with available CRISPR-based dependency screens, THP-1 and OCI-AML3 ranked second and third, respectively, in *NRAS* dependence (Figure 1A). Assessment of *KRAS* dependency ranked NB4 as the second-most *KRAS*-dependent AML cell line (Figure 1A). To these, we added two additional cell lines, HL-60 (*NRAS*-mutant) and ML-2 (*KRAS*-mutant). Full details of cell lines used in this study are provided in Figure S1A.

We next generated basal phospho-proteomic profiles for all five *RAS*-mutant cell lines. These data were first used to confirm RAS hyperactivation by using kinase set enrichment analysis (KSEA) to compare *RAS*-mutant cell lines with *RAS*^WT^ cell lines (MOLM-13, MV4;11 and PL-21). In line with RAS hyperactivation, this analysis showed significant enrichment of MAPK1 (ERK2) activity, as well as its downstream targets, CDKs (Figure 1B). Further, other RAS/RAF/MEK/ERK pathway members (MAPK3/ERK1, BRAF and MAPK6/ERK3) also showed modest albeit non-statistically significant enrichment in *RAS*-mutant cells (Figure 1B).

Based on the strong dependence of these AML cell lines on oncogenic RAS signaling, we set out to apply our structure-based model of RAS/RAF/MEK/ERK signaling, integrated with a physiologically based pharmacokinetic (PBPK) model of drug kinetics in mouse tissues, to *RAS*-mutant AML. This integrated model captures key intracellular processes, including ERBB receptor dimerization, recruitment of adaptor proteins to the plasma membrane, RAS activation through SOS1-mediated GDP-to-GTP exchange, RAF dimerization, MEK and ERK activation, and negative feedback loops from phosphorylated ERK to RAF, SOS1, and ERBB receptors. It also accounts for the kinetics of RAF inhibitors in mouse tissues and their allosteric interactions with various BRAF and CRAF protein complexes. The allosteric effects of conformation-specific drugs are quantitatively modeled using thermodynamic factors.^23^

While single-drug sensitivity can now be predicted *in silico* with semi-quantitative accuracy,^26^ the *in silico* prediction of drug synergy, currently addressed using regression-based and deep learning approaches, remains very limited, largely due to poor generalizability for novel drug combinations.^27^ Therefore, the primary objective of our integrated framework was to provide mechanistic, qualitative predictions of drug synergy and to explore the interplay between the PBPK and ERK pathway models, rather than seeking a point-by-point quantitative fit to specific experimental datasets.

The integrated model predicts the combinations of conformation-specific RAF inhibitors to be effective against *RAS*-mutant AML by durably and substantially suppressing ERK signaling. The predictions exploit the asymmetry of RAF dimers by targeting the most thermodynamically favorable conformation of each monomer before and after inhibitor binding. In line with previous data observed for *RAS*- and *RAF-*mutant solid tumors, modeling predicted two combination types as potentially effective in a leukemic context: Type I + Type II and Type I½ + Type II (Figures 1C and S1B).

### Combining Type I½/Type II RAF inhibitors is synergistic against NRAS and KRAS mutant AML *in vitro*

We first assessed the single-agent *in vitro* efficacy of RAF inhibitors representing both Type I½ (CO/DI, *n* = 5) and Type II (CI/DO, *n* = 3) inhibitor classes (Figure S2A). Inhibitors with the lowest average IC_50_ across all *RAS*-mutant AML cell lines were chosen as candidates for combination testing. Lifirafenib (BGB-283), TAK-632 and sorafenib were chosen as candidate Type II inhibitors, while encorafenib and vemurafenib were taken forward as representative Type I½ inhibitors. While all variations of Type I½/Type II combinations showed improved efficacy compared with single agents (Figures S2B and S2C), lifirafenib (Type II) and encorafenib (Type I½) were chosen for further experiments based on both observed synergy and clinical relevance, with encorafenib being FDA-approved and lifirafenib currently being evaluated in Phase II trials.

**Figure 2:**
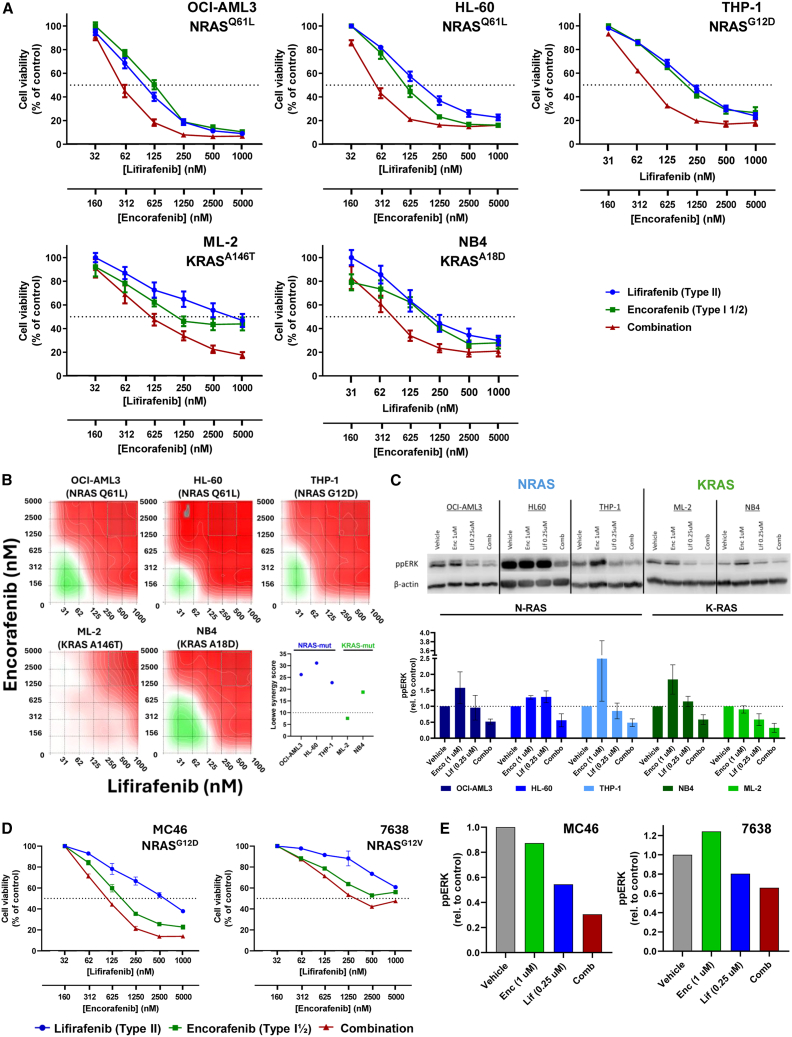
Combining type I½ + Type II RAF inhibitors is synergistic against *RAS*-mutant AML cell lines and patient samples *in vitro* (A) Cell viability in response to lifirafenib (Type II) + encorafenib (Type I½) compared with every single agent against five *RAS-*mutant AML cell lines. Cell viability was determined using AlamarBlue at 72 h post-treatment. Data points represent mean ± SEM from 3 replicate experiments. (B) Synergy plots for lifirafenib + encorafenib in all five cell lines tested. Synergy was determined using the Loewe Additivity method, calculated using the Synergy Finder web application (https://synergyfinder.fimm.fi/). A summary of synergy scores per cell line is included in the lower right panel. (C) Immunoblots showing relative levels of activated ERK (ppERK) in response to vehicle, single agent, and combination treatment (24 h) for all cell lines (top panel) with quantification shown in the lower panel. Quantification was performed using the mean ± SD from 3 biological replicates. (D) Cell viability in response to lifirafenib + encorafenib compared with each single agent against a primary *RAS-*mutant AML sample (MC46, left) and cells derived from a primary *RAS*-mutant AML PDX (7638, right). Data points represent either one (MC46) or three replicate experiments. (E) Quantification of immunoblots for MC46 (left) and 7638 (right) in response to vehicle, single agent or combination treatment (24 h) (*n* = 1). Images of immunoblots are provided in Figure S2D.

Both fixed-ratio and matrix (6 × 6) combinations were performed for all cell lines and synergy assessed using the Loewe Additivity method (>10 = synergy, 10 to −10 = additivity, <-10 = antagonism), chosen as both inhibitors share the same molecular target. In line with our structural biological predictions, this combination significantly reduced cell viability compared with single agents in all cell lines tested (Figure 2A). Strong synergy was observed in all *NRAS*-mutant cell lines, with Loewe Additivity scores of 31.1, 26.2 and 22.8 for HL-60, OCI-AML3 and THP-1, respectively. Synergy was also observed in the *KRAS*-mutant NB4 cell line (score = 18.7), while the combination was additive in the *KRAS*-mutant ML-2 cell line (score = 7.6) (Figure 2B). To assess the robustness of the observed synergy and the Loewe additivity method, we evaluated our results using two alternative synergy metrics (HSA; highest single agent and BLISS Additivity). These results were concordant with Loewe Additivity scores (Table S1).

We next assessed the effect of the combination on pathway activity via measurement of double phosphorylated ERK (ppERK, which we have shown correlates strongly with cell viability^16^) 24 h post-drug treatment. In line with our cell viability results, ppERK was significantly reduced in all cell lines following combination treatment, as compared with single-agent and vehicle-treated cells. Levels of ppERK in combination-treated cells were reduced to 52%, 56%, 49%, 58% and 32% of control in OCI-AML3, HL-60, THP-1, NB4 and ML-2 lines, respectively (Figure 2C). Assessment of ppERK levels also highlighted the potential for paradoxical activation with the use of single RAF inhibitors, with increased pathway activity observed in 4/5 cell lines in response to encorafenib, and 2/5 cell lines following lifirafenib treatment (Figure 2C).

Next, we assessed the efficacy of the combination against two patient-derived *NRAS*-mutant AML samples *ex vivo* (sample details provided in Figure S2D). These represented either primary AML cells (MC46, *NRAS*^G12D^) or cells derived from NSGS mice following the primary *in vivo* expansion of patient material (7638, *NRAS*^G12V^). In both cases, the combination was more effective than either single agent (Figure 2D), with Loewe Additivity scores of −1.4 and −1.53 for MC46 and 7638, respectively. In line with the decrease in viability, we also observed a decrease in ppERK levels in response to combination treatment compared with single-agent and vehicle controls (Figures 2E and S2E).

In order to benchmark the efficacy of this combination against direct RAS inhibitors, all five cell lines were also treated with BI-2852 (KRAS switch I/II pocket inhibitor) and BI-3406 (KRAS:SOS1 interaction inhibitor). We observed little to no effect on viability, with BI-3406 failing to reach an IC_50_ in any cell line at 10 μM and BI-2852 achieving an IC_50_ in only 1/5 cell lines (NB4, 7.7 μM) (Figure S2F).

### Combining Type I/Type II RAF inhibitors is synergistic against NRAS-mutant AML *in vitro*

**Figure 3:**
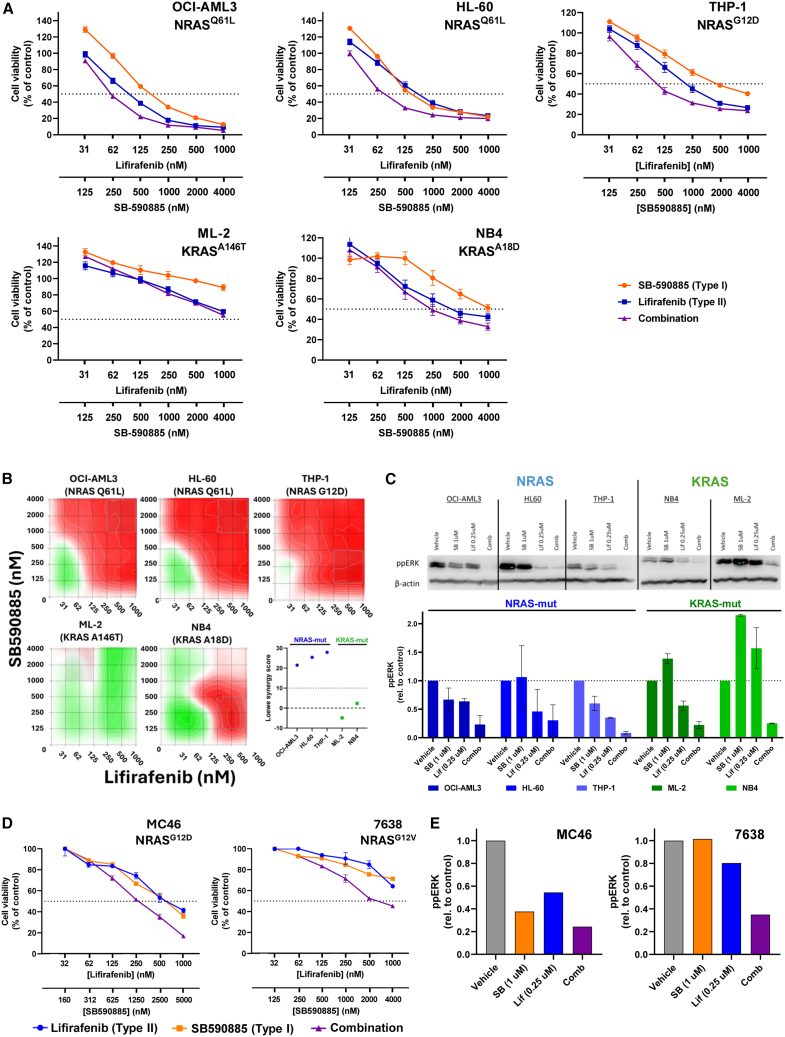
Combining **Type I + Type** II RAF inhibitors is synergistic against *NRAS*-mutant AML cell lines and patient samples *in vitro* (A) Cell viability in response to lifirafenib (type II) + SB590885 (type I) compared with each single agent against five *RAS-*mutant AML cell lines. Cell viability was determined using AlamarBlue at 72 h post-treatment. Data points represent mean ± SEM from 3 replicate experiments. (B) Synergy plots for lifirafenib + SB590885 in all five cell lines tested. Synergy was determined using the Loewe Additivity method, calculated using the Synergy Finder web application (https://synergyfinder.fimm.fi/). A summary of synergy scores per cell line is included in the lower right panel. (C) Immunoblots showing relative levels of activated ERK (ppERK) in response to vehicle, single agent, and combination treatment (24 h) for all cell lines (top panel) with quantification shown in the lower panel. Quantification was performed using the mean ± SD from 3 biological replicates. (D) Cell viability in response to lifirafenib + SB590885 compared with each single agent against a primary *RAS-*mutant AML sample (MC46, left) and cells derived from a primary *RAS*-mutant AML PDX (7638, right). Data points represent either one (MC46) or three replicate experiments. (E) Quantification of immunoblots for MC46 (left) and 7638 (right) in response to vehicle, single agent, or combination treatment (24 h) (*n* = 1). Images of immunoblots are provided in Figure S3B.

As with the previous combination, we first assessed the single-agent efficacy of Type I inhibitors to guide the selection of combinations to be tested. Only two Type I inhibitors are currently available, SB590885 and GDC-0879. Given the superior single-agent efficacy of SB590885 across all cell lines (Figure S3A), this was chosen for combination testing alongside the Type II inhibitor lifirafenib. This combination significantly reduced cell viability compared with single-agent treatments in all *NRAS*-mutant cell lines but showed only modest or no improvement in *KRAS*-mutant lines (Figure 3A). This was reflected in the assessment of synergy, with Loewe Additivity scores for *NRAS*-mutant cell lines equating to strong synergy (scores = 27.9, 25.4 and 21.5 for THP-1, HL-60 and OCI-AML3, respectively). Loewe Additivity scores for *KRAS*-mutant lines indicated additivity (scores = 2.4 and −4.9 for NB4 and ML-2, respectively) (Figure 3B). Assessment using alternative synergy metrics (HSA and BLISS Additivity) was in line with Loewe Additivity data (Table S2).

As with our previous results, synergy in *NRAS*-mutant cell lines was reflected in the suppression of ERK pathway activity. Combination treatment caused a reduction of ppERK levels to 23%, 30% and 8% of control for OCI-AML3, HL-60 and THP-1 cells, respectively (Figure 3C). Interestingly, the ERK pathway activity was also significantly decreased in KRAS-mutant cell lines despite the lack of synergy observed for the combination in cell viability assays.

In line with the combination efficacy observed in *NRAS*-mutant cell lines, this combination also showed an additive effect against the two patient-derived samples MC46 and 7638 (Figure 3D), with Loewe Additivity scores of −3.37 and −2.14, respectively. Consistent with the *ex vivo* efficacy experiments, we again observed considerable reduction in ppERK levels in the combination-treated cells compared to single-agent and vehicle treatments (Figures 3E and S3B).

### Combined Type I and Type II RAF inhibitors show site-specific efficacy in aggressive RAS-mutant AML *in vivo*

**Figure 4:**
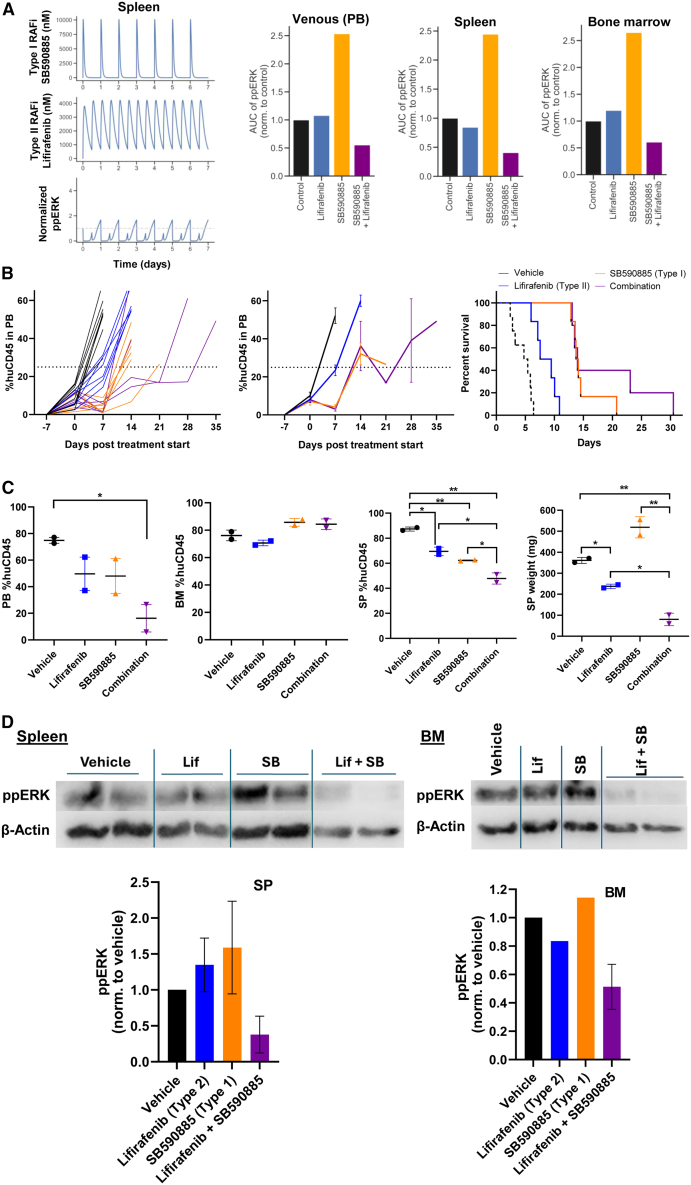
Combining Type I + Type II RAF inhibitors shows site-specific efficacy against an aggressive *NRAS*-mutant AML PDX *in vivo* (A) *In silico* prediction of phosphorylated ERK (ppERK) levels in response to treatment was determined by our integrated physiologically based pharmacokinetic (PBPK) and structure-based modeling approach. Inhibitor concentrations are given in nanomolar (nM), ppERK levels are normalized to the levels in the untreated condition. The left panel shows time course predictions of ppERK levels in the spleen over 7 days (see Figure S4A for ppERK level predictions in peripheral blood [PB] and bone marrow [BM]). The right panels display the area under the curve (AUC) of ppERK levels for all treatment groups across PB, spleen, and BM. (B) Response of AML005 to vehicle, lifirafenib, SB590885, and combination treatment as determined by percent chimerism of mouse versus human CD45^+^ cells in peripheral blood (PB). The percentage of hCD45^+^ cells is shown for individual mice (left) and for median values for each treatment group (middle). Event-free survival (event = 25% hCD45^+^ cells in PB) is shown for all treatment groups (right). (C) Data from two representative mice euthanized at Day 10 post-treatment initiation showing leukemia burden by %hCD45^+^ cells in PB, bone marrow (BM, femur + tibia) and spleen (SP), and by spleen weight for all treatment groups. (D) Images and quantification of immunoblots of Day 10 spleen (left) and BM (right) samples. Quantification was based on 2 biological replicates where sufficient material was available (spleen and Lif + SB BM). Otherwise, quantification reflects one biological sample. Statistical significance was assessed using unpaired two-tailed t tests, indicated by asterisks (∗*p* < 0.05 and ∗∗*p* < 0.01).

We developed an *in silico* PBPK model that calculates drug concentrations at different anatomical sites for each specific RAFi and its treatment regimen. Output from the PBPK model was integrated with our existing structure-based model of MAPK signaling (see Data S1).^28^ This combined model enabled prediction of ppERK time courses following treatment with Type I and Type II RAF inhibitors, administered either individually or in combination. Figure 4A shows the predicted time courses of the Type I RAFi SB590885 and the Type II RAFi lifirafenib, along with the resulting ppERK levels in spleen; corresponding profiles for PB and BM are shown in Figure S4A. The model predicts that combination treatment effectively suppresses ppERK in BM, spleen, and circulating leukemia blasts (peripheral blood; PB), despite the strong paradoxical ERK activation observed when SB590885 is used alone (Figures 4A and S4A).

To assess the robustness of the model outcomes, we performed a sensitivity analysis in which each model parameter was individually perturbed and resulting changes in the cumulative ppERK response - our primary model output - quantified across venous blood, spleen, and BM (Figure S4B). The predicted ppERK responses are highly robust to variation in site-specific PBPK parameters, including tissue volumes (V) and blood flow rates (Q). This suggests that the modeled qualitative signaling predictions are not strongly driven by uncertainties in compartment geometry or perfusion but instead are governed by biochemical properties of the signaling proteins and inhibitors involved. Conversely, ppERK responses exhibit the highest sensitivity to biochemical parameters directly controlling pathway activation, most notably equilibrium dissociation constants (Kd) of RAF inhibitors for BRAF, as well as kinetic parameters governing the RAF activation cycle and GAP-mediated deactivation of mutant and wild-type RAS isoforms. This behavior is consistent with our previous analyses and mechanistic understanding of MAPK pathway regulation^16^^,^^28^ and supports the interpretation that these parameters represent true biological control points rather than model-specific artifacts.

To validate RAFi combinations in a clinically relevant preclinical model, we first assessed the *in vivo* efficacy of lifirafenib + SB590885 against AML005, an aggressive AML patient-derived xenograft (PDX) harboring a *KMT2A*:*:MLLT4* fusion with an activating *NRAS*^G12A^ mutation. Doses and schedules for both drugs were chosen based on previously published data in immune-deficient mouse strains.^29^^,^^30^ Given the reported instability of *RAS* mutation frequencies, the presence of the *NRAS* mutation was confirmed between primary and secondary *in vivo* passages (Figure S4C). In line with the aggressiveness of this PDX, all cohorts exhibited high leukemia burden in PB at commencement of treatment (group median range 7.2–11.1% huCD45^+^ cells in PB).

Contrary to *in vitro* data, SB590885 was more effective than lifirafenib as a single agent and showed similar efficacy to the combination treatment. Disease regression, as measured by %huCD45+ cells in PB, was observed in 5/8 mice for both the SB590885 and combination groups following one week of treatment (Figure 4B). Of the evaluable six SB590885 mice remaining after Day 10, five reached event by the end of the second week of treatment (median Leukemia Growth Delay; LGD = 8.5 days), with one animal achieving an LGD of 15 days. While the lifirafenib + SB590885 combination proved tolerable in earlier tolerability testing performed in naive mice (Figure S4D), we did observe adverse treatment effects in 2/6 mice, prompting removal of one animal from the study. Of the five remaining evaluable mice (post Day 10), three reached event by the end of the second week of treatment (median LGD = 8.4 days), with the remaining two animals achieving an LGD of 18 days and 25.4 days (Figure 4B). Median LGD values for all treatment groups are provided in Figure S4E.

To assess therapeutic enhancement, or *in vivo* synergy, we applied a statistical threshold of *p* = 0.05 in comparing median EFS for the combination group versus single treatment cohorts. Both single-agent SB590885 and the combination significantly improved survival compared with lifirafenib (*p* = 0.004 and *p* = 0.01, respectively). Despite the increased LGD observed for two mice in the combination group, there was no significant difference in survival compared with SB590885 alone (*p* = 0.28) and therefore no therapeutic enhancement.

Two representative mice from each cohort were euthanized at Day 10 post-treatment initiation to assess leukemia burden in the spleen and bone marrow (BM). Assessment of PB showed that SB590885 + lifirafenib more effectively cleared circulating blasts compared with SB590885 alone, though this was not statistically significant (*p* = 0.197, Figure 4C). We observed a marked difference between the two groups in their effect on spleen-resident PDX cells at Day 10. All treatments exhibited significantly lower percentages of huCD45+ cells in the spleen, with the combination group having a significantly lower percentage of blasts compared with SB590885 alone (*p* = 0.045, Figure 4C). Notably, the spleens from combination mice were significantly smaller (median = 80 mg, 78% decrease vs. vehicle) than all other groups, particularly the SB590885-treated mice, which had considerably enlarged spleens (median = 519 mg, 43% increase vs. vehicle) (Figure 4C). These results suggest that while both SB590885 and the SB590885 + lifirafenib combination effectively clear circulating blasts from the PB, the combination potently targets leukemia residing in the spleen.

We next performed immunoblotting on Day 10 spleen and BM samples to determine the effect of each treatment on RAS/RAF/MEK/ERK signaling, measured by ERK phosphorylation (ppERK), in different anatomical niches. In line with spleen weights, we observed a reduction in ppERK levels in spleen-derived cells from combination-treated mice to 37% of vehicle controls (Figure 4D). We also observed paradoxical activation of ppERK in the spleens of mice treated with either single agent, which was more pronounced in SB590885-treated samples (Figure 4D).

Despite the efficacy observed in spleen samples, the combination proved ineffective at clearing leukemia from the BM. No significant differences were observed in BM %huCD45+ cells for any treatment group compared with the vehicle-treated cohort (Figure 4B). Notably, ppERK levels in the BM of combination-treated mice were reduced to 51% of vehicle control levels (Figure 4D), suggesting that the BM niche provides a protective environment against the inhibition of RAS/RAF/MEK/ERK signaling.

### Combining type I½ and ***T***ype II RAF inhibitors improves survival of an aggressive RAS-mutant AML *in vivo*

**Figure 5:**
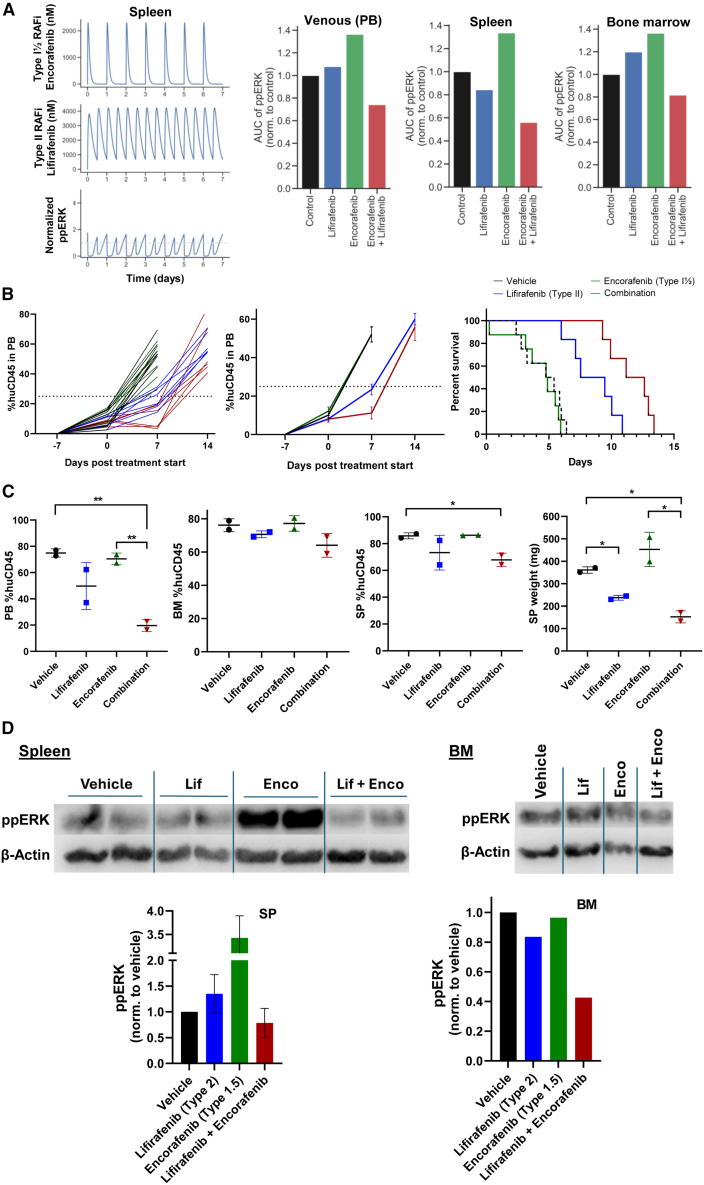
Combining Type I½ + Type II RAF inhibitors synergistically improves survival of an aggressive *NRAS*-mutant AML PDX *in vivo* (A) *In silico* prediction of phosphorylated ERK (ppERK) levels in response to treatment was determined by our integrated physiologically based pharmacokinetic (PBPK) and structure-based modeling approach. Inhibitor concentrations are expressed in nanomolar (nM), ppERK levels are normalized to the level in the untreated condition. The left panel shows time course predictions of ppERK levels in the spleen over 7 days (see Figure S5A for ppERK level predictions in peripheral blood [PB] and bone marrow [BM]). The right panels display the area under the curve (AUC) of ppERK levels for all treatment groups across PB, spleen, and BM. (B) Response of AML005 to vehicle, lifirafenib, encorafenib, and combination treatment as determined by percent chimerism of mouse versus human CD45^+^ cells in peripheral blood (PB). The percentage of hCD45^+^ cells is shown for individual mice (left) and for median values for each treatment group (middle). Event-free survival (event = 25% hCD45^+^ cells in PB) is shown for all treatment groups (right). (C) Data from two representative mice euthanized at Day 10 post-treatment initiation showing leukemia burden by %hCD45^+^ cells in PB, bone marrow (BM, femur + tibia) and spleen (SP), and by spleen weight for all treatment groups. (D) Images and quantification of immunoblots of Day 10 spleen (left) and BM (right) samples. Quantification was based on 2 biological replicates where sufficient material was available (spleen). Otherwise, quantification reflects one biological sample. Statistical significance was assessed using unpaired two-tailed t tests, indicated by asterisks (∗*p* < 0.05 and ∗∗*p* < 0.01).

As in the previous section, we first applied our integrated model to calculate the time courses of ppERK levels in AML cells, this time following treatment with Type I½ (encorafenib) and Type II (lifirafenib) RAF inhibitors, administered either individually or in combination. The analysis was conducted in PB, spleen, and BM (Figures 5A; left panel; S5A). As before, we also calculated the corresponding areas under the time course curves for each condition (Figure 5A, right panels). For the lifirafenib + SB590885 combination, our integrated PBPK/structure-based model predicted a synergistic reduction in ppERK levels in AML cells across BM, spleen, and PB (Figures 5A and S5A).

We next assessed the *in vivo* efficacy of lifirafenib in combination with encorafenib in the same PDX model. Inhibitor doses and schedules were again based on previously published data.^31^ Results of tolerability testing for this combination are shown in Figure S5B.

Despite the high level of engraftment at the beginning of treatment, 2/8 combination-treated mice achieved leukemia regression at Day 7 compared with Day 0, while regression was not observed in response to either single agent (Figure 5B). The combination achieved a significantly greater LGD (6.83 days) compared with either lifirafenib (4.39 days, *p* = 0.019) or encorafenib (−0.25 days, *p* < 0.001) alone (Figure S5C). Using the threshold of *p* < 0.05, the combination group achieved therapeutic enhancement as the EFS for combination-treated mice was significantly greater than that of lifirafenib-treated (*p* = 0.015) and encorafenib-treated (*p* < 0.001) cohorts (Figure 5B).

In line with the PB data, encorafenib-treated mice showed equivalent engraftment levels to vehicle-treated mice in both BM and spleen at Day 10. Comparing the combination with lifirafenib alone showed only a modest decrease in the percentage of huCD45+ cells in BM and spleen, despite the combination group exhibiting considerably fewer circulating blasts at Day 10 (Figure 5C). Interestingly, increased clearance of spleen-resident PDX cells by the combination, as compared with lifirafenib alone, was more evident when assessing raw spleen weight (Figure 5C).

We observed considerable activation of ppERK in the spleen-derived cells of encorafenib-treated mice when compared with the control cohort (Figure 5D), which was in line with the increased spleen weight. Lifirafenib-treated mice showed a slight increase in ppERK levels in the spleen compared with controls, despite the reduction observed in %huCD45+ cells and spleen weight (Figures 5C and 5D). This might be explained by a paradoxical pathway activation by a single RAF inhibitor. Compared with vehicle- and single agent-treated groups, spleen samples from combination-treated mice exhibited a mild reduction in ppERK levels. This was in line with the reduction observed in measurements of blast percentage and weight from spleen samples. Day 10 samples from BM showed a strong reduction in ppERK following treatment with the combination (to 42% of vehicle levels). Surprisingly, we observed a 50% reduction in ppERK signal for the combination compared with lifirafenib treatment despite there being only a mild difference in %huCD45+ cells in the BM (Figures 5C and 5D). As we observed a similar decrease in ppERK levels in response to the lifirafenib + SB590885 combination (Figure 4D), it is possible that targeting RAS/RAF/MEK/ERK signaling in the BM is not sufficient to cause cell death due to compensatory factors in the BM niche. Nevertheless, these results suggest that the lifirafenib + encorafenib combination provides marked benefits in therapeutic effect and tolerability when compared with the equivalent lifirafenib + SB590885 treatment.

## Discussion

In this study, we showed that dual targeting of RAF by combining two discrete, conformation-specific RAF inhibitors is a promising strategy for treating *RAS*-mutant AML. Using our refined, AML-focused model of RAS/RAF/MEK/ERK signaling, we predicted that co-inhibition of RAF using either Type I½ + Type II or Type I + Type II inhibitor combinations would synergistically decrease pathway activity. These predictions were first validated in *in vitro* models representing both *NRAS*- and *KRAS*-mutant AML. Importantly, both combination types proved effective against primary *NRAS*-mutant AML samples *ex vivo* and an aggressive pediatric *NRAS*-mutant AML PDX model.

To position our findings within the broader therapeutic landscape, MAPK-targeted strategies for RAS-mutant AML can be broadly categorized into three mechanistic approaches: downstream inhibition (MEK/ERK), direct RAS inhibition, and RAF-based combinations. Despite the potential benefit of inhibiting ERK pathway activation in AML, downstream strategies relying on MEK inhibitors (without concomitant RAF inhibition) have yielded disappointing clinical outcomes, showing only modest efficacy.^32^^,^^33^ These approaches are largely limited by a brief durability of response and dose-limiting toxicities.^11^^,^^34^^,^^35^^,^^36^ While terminal-node ERK inhibitors are currently being explored as an alternative downstream strategy, they face similar challenges related to the therapeutic window and toxicity. Further upstream, inhibitors directly targeting RAS hold promise, though the clinical use of earlier compounds has been limited to specific patient subsets given their strict affinity for defined point mutations (e.g., KRAS^G12D^). However, this therapeutic landscape is continuing to evolve with the emergence of direct, multi-selective RAS(ON) inhibitors (e.g., RMC-7977), which are currently demonstrating promising preclinical efficacy in combination regimens.^37^

While RAF inhibitors are initially effective in cancers harboring mutant BRAF (V600E), single-agent RAF inhibition in RAS-mutant cancers has been approached with caution due to reports of diminished efficacy or paradoxical ERK activation upon the engagement of wild-type RAF. Investigation of RAF inhibition in RAS-mutant AML has included the pan-RAF inhibitors LY3009120 and belvarafenib, both of which demonstrated only modest single-agent activity.^38^^,^^39^ To overcome this, different combination strategies were used, focused either no combining RAF and MEK inhibitors,^40^ or combining structurally different RAF inhibitors.^16^^,^^28^^,^^41^ We have previously shown, both theoretically and experimentally, that although both dual-RAF combinations and RAF plus MEK combinations can achieve synergy in RAS-mutant AML cells, dual-RAF inhibition provides a broader synergy window and a significantly reduced propensity for paradoxical ERK activation compared to the RAF plus MEK approach.^41^ This distinction has direct clinical relevance: Pharmacokinetic clearance inevitably produces transient periods of sub-therapeutic drug exposure (as illustrated in Figures 4A and 5A), and minimizing paradoxical activation across this concentration range is critical for reducing the overall ppERK area under the curve. Dual-RAF inhibitor combinations have furthermore demonstrated acceptable tolerability in clinical studies, underscoring their translational potential.^42^^,^^43^ To our knowledge, the present study is the first to explore dual RAF inhibition in blood cancers, and in doing so we provide a novel, non-obvious, and effective therapeutic strategy for treating RAS-mutant AML.

A major consideration in the use of inhibitors targeting RAS/RAF/MEK/ERK signaling is the potential for paradoxical activation of downstream signaling, particularly when targeting RAF. This phenomenon results from ligand-induced dimerization and allosteric modulation of the opposing (unbound) monomer to increase kinase activity.^44^^,^^45^ Following *in vitro* treatment, paradoxical activation was observed in response to encorafenib and SB590885 when used as single agents. Importantly, in all cases both combination types ablated this effect to synergistically reduce ppERK levels, which is in line with our previous observations in solid tumor models.^16^^,^^24^ While the reduction in ppERK levels largely correlated with cell viability in response to both combinations, this was not observed for the lifirafenib + SB590885 combination against *KRAS-*mutant cell lines. Similar effects of RAFi combinations on ppERK levels and cell viability were previously observed in the KRAS-mutant PANC1 pancreatic cancer cell line, suggesting plasticity of growth signaling in these cancer cells.^28^ However, this discrepancy between ppERK levels and cell viability was not observed in response to lifirafinib + encorafenib.

While lifirafenib + SB590885 showed considerable efficacy *in vivo* against AML005, the combination did not significantly improve EFS compared with single-agent SB590885. However, the assessment of leukemia burden in the spleens of representative mice at Day 10 showed significantly greater clearance of blasts in response to combination treatment. This was most pronounced in the measurement of spleen weight, where combination treatment decreased weight to approximately the size of non-engrafted immune-deficient mice.^46^^,^^47^ Conversely, SB590885 alone increased the spleen weight compared with control mice. While SB590885 treatment appeared to reduce the percentage of hCD45^+^ cells in the spleen compared with controls, the significantly increased spleen size suggests that the number of blasts had increased. This may be explained by the increase in ppERK levels observed in the spleens of SB590885-treated mice and provides important context for the benefit of combining Type I and Type II inhibitors despite observing similar EFS based on PB values. While our data suggest that Type I + Type II RAFi combinations warrant further investigation, translational relevance of Type I inhibitors is currently limited. To date, no Type I inhibitor has progressed beyond the preclinical stage, and use of these inhibitors as single agents causes strong paradoxical activation of downstream signaling in *RAS*-mutant/*RAF*-WT cancers.^14^ It is noteworthy that while EFS was not significantly different between combination- and SB590885-treated groups, both treatments achieved a significant delay in disease progression. Therefore, future studies should focus on the combination as a way to optimize Type I inhibitor use by preventing paradoxical activation.

Importantly, the Type I½ and Type II inhibitors used in this study are either under active clinical investigation (lifirafenib) or FDA-approved (encorafenib). This combination showed significantly greater efficacy compared to either single agent, which is striking in the context of a highly aggressive PDX model. As a result of the rapid expansion of AML005 in NSGS mice, the leukemia burden across all cohorts was significantly higher at treatment initiation than the intended ≥1% hCD45^+^ cells in PB. Interestingly, despite observing only a modest decrease in %hCD45^+^ cells in BM upon combination treatment, ppERK levels were significantly reduced. This suggests that these agents reach the BM but the effect of RAF inhibition on cell viability is diminished. While further investigation is required to understand this discrepancy, we hypothesize that microenvironmental factors may play a protective role in response to RAF inhibition. One explanation for this in this specific model might be the supraphysiological levels of stem cell factor (SCF) in NSGS mice.^48^ Binding of SCF to its cognate receptor, c-KIT, can activate signaling pathways such as PI3K-mTOR, which can compensate for RAS/RAF/MEK/ERK signaling.^49^ Existing literature suggests that the hypoxic nature of the BM microenvironment might also be a contributing factor. It has been reported that parallels exist between hypoxia and the consequences of RAS activation, with both exhibiting induction of fatty acid scavenging.^50^ This overlap may increase the permissiveness of the BM microenvironment for the growth of *RAS*-mutant AML. We do, however, stress that these hypotheses require experimental validation to determine how these factors might have affected our observed results, and whether this has any implications for any future therapeutic implementation of these strategies.

Future investigations into either combination type may benefit from the modulation of doses and schedules used *in vivo*. Predictions of the effect of each combination using our integrated PBPK/structure-based model were corroborated by experimental data from mice evaluated at Day 10 during treatment. However, the incorporation of experimental data into our model would refine predictions to inform more precise dosing schedules. Additionally, the synergistic effects of dual RAF inhibition may benefit from the addition of agents targeting parallel signaling pathways, such as PI3K inhibitors.

Together, our data suggest that dual targeting of RAF using conformation-specific inhibitors is an effective, well-tolerated strategy for treating *RAS-*mutant AML. These results provide a platform for refining RAFi combinations for clinical use, and a proof-of-concept that may be applied to other hematological malignancies with activated RAS signaling.

### Limitations of this study

*In vitro* results described in this paper come from experiments in AML cell lines. While these lines harbor RAS mutations and have been shown to depend on RAS pathway activation, these cell lines feature diverse genetic backgrounds that may directly or indirectly impact the activity of RAS and other signaling pathways. This limitation was mitigated by testing across a diverse range of cell lines, but further investigations may benefit from testing RAFi combinations in complementary model systems. The evaluation of our RAFi combinations in one PDX model *in vivo* also presents a limitation in interpreting the translational benefit of our findings. Efficacy testing in an expanded panel of RAS-mutant AML PDXs would significantly increase understanding of the potential clinical utility of our combinations.

## Resource availability

### Lead contact

Requests for further information and resources should be directed to and will be fulfilled by the lead contact, Prof. Jonathan Bond (jonathan.bond@ucd.ie).

### Materials availability

This study did not generate new unique reagents.

### Data and code availability

• Data: Mass spectrometry proteomics data have been deposited to the ProteomeXchange Consortium via the PRIDE partner repository with the dataset identifier PXD063606.
• Code: This paper does not report original code.
• Other items: Any additional information required to reanalyze the data reported in this paper are available from the lead contact upon request.

## Acknowledgments

The authors would like to thank the members of the Patient-Derived Xenograft Core at the Baylor College of Medicine, TX, USA, who kindly provided the AML-005 sample used in this study. We would also like to thank Dr. Ronald Stam and Susan Arentsen-Peters from the Princess Maxima Center for Pediatric Oncology, Utrecht, The Netherlands, for kindly providing the 7638 sample. Additionally, we thank the team at VIVO Biobank (UK). The MC46 sample and associated data used in this study were provided by VIVO Biobank, supported by 10.13039/501100000289Cancer Research UK & 10.13039/501100015570Blood Cancer UK (grant no. CRCPSC-Dec21\100003). The authors thank the 10.13039/501100001593Irish Cancer Society, which fully supported LJ throughout this project through a Translational Research Fellowship (CRF20JON). The authors would also like to thank the 10.13039/100014364National Children's Research Centre for their significant support of this work through Leadership Award A-18-3 that funded start-up research in the Bond laboratory at Systems Biology Ireland. JB is supported by Research Ireland (20/FFP-P/8844). TD is supported by 10.13039/100031870Children's Health Ireland and Research Ireland for the Precision Oncology Ireland program (18/SPP/3522). BK is supported by the 10.13039/100000002National Institutes of Health (10.13039/100000002NIH; R01CA244660, R01HL171773) and the 10.13039/501100000780European Union (101136926 MULTIR). OR is supported by Research Ireland (22/PATH-S/10875). CC is supported by the Research Ireland SFI Centre for Research Training in Genomics Data Science (18/CRT/6214). Proteomics services are supported by 10.13039/501100025294Research Ireland (SFI Research Infrastructure Programme, 18/RI/5702). The funders played no role in study design, data collection, analysis and interpretation of data, or the writing of this manuscript. Additionally, the authors thank and acknowledge the many team members at Systems Biology Ireland and the Irish National Children’s Cancer Service at Children’s Health Ireland at Crumlin for their support and helpful suggestions during this project.

## Author contributions

Conceptualization: L.J., J.B., and B.K. methodology: O.R., H.I., K.W., and B.K. investigation: L.J., O.R., T.D., H.I., and C.C. visualization: L.J. funding acquisition: L.J. and J.B. project administration: L.J. supervision: J.B. and B.K. writing – original draft: L.J. and J.B. writing – review and editing: L.J., O.R., T.D., H.I., C.C., K.W., B.K., and J.B.

## Declaration of interests

O.R. and B.K. filed a patent application (WO2019224216A1) on inhibitor combinations to inhibit kinases whose activation includes dimerization or oligomerization. All other authors declare they have no competing interests.

## STAR★Methods

### Key resources table

**Table:** 

REAGENT or RESOURCE	SOURCE	IDENTIFIER
**Antibodies**		

FITC anti-mouse CD45	Biolegend	RRID:AB_312973
APC anti-human CD45	Biolegend	RRID:AB_2562049
FITC Rat IgG2b, κ Isotype Ctrl	Biolegend	RRID:AB_326550
APC Mouse IgG1, κ Isotype Ctrl	Biolegend	RRID:AB_2888687
Anti-MAP Kinase (ERK-1, ERK-2) antibody produced in rabbit	Sigma-Aldrich	RRID:AB_477216
Monoclonal Anti-MAP Kinase, Activated (Diphosphorylated ERK-1&2) antibody produced in mouse	Sigma-Aldrich	RRID:AB_477245
β-Actin (13E5) Rabbit mAb	Cell Signaling Technologies	RRID:AB_2223172

**Biological samples**		

Patient-derived xenograft (AML005)	Baylor School of Medicine PDX Core	https://pdxportal.research.bcm.edu/pdxportal
MC46 (primary leukemia cells)	VIVO Biobank (UK)	https://vivobiobank.org/
7638 (primary leukemia cells)	Princess Maxima Center for Pediatric Oncology	N/A

**Chemicals, peptides, and recombinant proteins**		

RBC Lysis/Fixation Solution (10*X*)	Biolegend	Cat# 422401
GDC-0879	Selleck Chemicals	Cat# S1104
Vemurafenib	Selleck Chemicals	Cat# S1267
Dabrafenib	Selleck Chemicals	Cat# S2807
PLX7904	Selleck Chemicals	Cat# S7964
Sorafenib	Selleck Chemicals	Cat# S7397
TAK-632	Selleck Chemicals	Cat# S7291
Tovorafenib/TAK-580	Selleck Chemicals	Cat# S7121
RAF709	Selleck Chemicals	Cat# S8690
RAF265	Selleck Chemicals	Cat# S2161
Lifirafenib	MedChem Express	Cat# HY-18957
Encorafenib	MedChem Express	Cat# HY-15605
SB590885	MedChem Express	Cat# HY-10966
Tween-80	Merck	Cat# P1754-500 ML
*N-N*-dimethylacetamide	MedChem Express	Cat# HY-W042416
Cremophor EL	SelleckChem	Cat# S6828
Carboxymethyl cellulose	Merck	Cat# C5678-500G
Resazurin Sodium Salt	Merck	Cat# 199303
Methylene Blue	Sigma-Aldrich	Cat# M9140
Potassium hexacyanoferrate (III)	Sigma-Aldrich	Cat# 244023
Potassium hexacyanoferrate	Sigma-Aldrich	Cat# 455989

**Deposited data**		

Mass spectrometry proteomics data	PRIDE database, https://www.ebi.ac.uk/pride/	Dataset Identifier PXD063606

**Experimental models: Cell lines**		

OCI-AML3	DSMZ	RRID:CVCL_1844
HL60	DSMZ	RRID:CVCL_0002
THP-1	DSMZ	RRID:CVCL_0006
NB4	DSMZ	RRID:CVCL_0005
ML-2	DSMZ	RRID:CVCL_1418

**Experimental models: Organisms/strains**		

Mouse: NSGS (NOD-scid IL2Rgnull-3/GM/SF)	Charles River Laboratories (UK)	RRID:IMSR_JAX:013062

**Oligonucleotides**		

Primer for NRAS Exon 2 (FWD); 5′-GCTCGCCAATTAACCCTGATTAC-3′	This paper/Eurofins Genomics	N/A
Primer for NRAS Exon 2 (REV); 5′-TGGGTAAAGATGATCCGACAAGTGA-3′	This paper/Eurofins Genomics	N/A

**Software and algorithms**		

GraphPad Prism (v8.0.2)	GraphPad	RRID:SCR_000306
Perseus 1.6.15.0	http://www.perseus-framework.org	RRID:SCR_015753
MaxQuant (v2.0.1.0)	http://www.biochem.mpg.de/5111795/maxquant	RRID:SCR_014485
Kinase Set Enrichment Analysis (KSEA; v0.99.0)	https://github.com/casecpb/KSEA	N/A
Jupyter Notebook	https://jupyter.org/	RRID:SCR_018315
R Project for Statistical Computing (v4.4.1)	http://www.r-project.org/	RRID:SCR_001905
SynergyFinder Web Application (v3.0)	https://synergyfinder.fimm.fi/	RRID:SCR_019318
SoftMax Pro (v6.2.2)	Molecular Devices	RRID:SCR_014240
Image Studio Lite	http://www.licor.com/bio/products/software/image_studio_lite/	RRID:SCR_013715
Python 3.12	https://www.python.org	RRID:SCR_008394
PySB (v1.16)	https://pysb.org	N/A
BioNetGen (v2.9.2)	https://bionetgen.org	N/A

**Other**		

Phusion High-Fidelity DNA Polymerase	New England Biolabs	M0530S

### Experimental model and study participant details

#### Patient-derived xenograft (PDX) models

All *in vivo* experiments in this study were performed using NOD-scid IL2Rgnull-3/GM/SF (NSGS) mice. Breeding pairs were purchased from Charles River Laboratories, with breeding and colony maintenance undertaken in-house in the Biomedical Facility (BMF) at University College Dublin. Male and female progeny were used in equal number for all *in vivo* experiments. For drug tolerability and efficacy experiments, experimental groups contained an equal distribution of male and female mice. For this, animals of the same sex were randomly allocated to experimental groups. There was no observable influence of sex on engraftment rates, drug tolerability or treatment response. All mice were maintained under specific-pathogen-free (SPF) conditions within the BMF.

All animal experiments were approved by the UCD Animal Research Ethics Committee (Approval AREC-21-04-Bond) and the Health Products Regulatory Authority (HPRA; Authorisation AE18982/P199).

#### Cell lines and primary culture

Cell lines were acquired from American Type Culture Collection (ATCC) or Deutsche Sammlung von Mikroorganismen und Zellkulturen (DSMZ). HL-60 (female), OCI-AML3 (male), THP1 (male) and NB4 (female) cells were cultured in RPMI 1640 supplemented with GlutaMAX (2 mM) and 10% fetal bovine serum. ML-2 (male) cells were cultured RPMI 1640 with GlutaMAX (2 mM) and 20% fetal bovine serum. All cell lines were cultured at 37°C and 5% CO2. The identities of all cell lines were validated using the cell line authentication service provided by Eurofins genomics (https://eurofinsgenomics.eu/en/genotyping-gene-expression/applied-genomics-services/cell-line-authentication/), using the Applied BiosystemsTM AmpFLSTRTM IdentifilerTM Plus PCR Amplification Kit system. Cell lines in culture were tested regularly (at least every 3 months) for mycoplasma using Lonza’s MycoAlert Mycoplasma Detection Kit (LT07-710) according to the manufacturer’s instructions.

For *ex vivo* studies, primary leukemia (MC46; male) and primary PDX cells (7638; female) were cultured RPMI 1640 with GlutaMAX (2 mM) and 20% fetal bovine serum. Primary and PDX cells were maintained at 37°C and 5% CO2 for the duration of *ex vivo* experiments.

### Method details

#### Inhibitors

For *in vitro* use, all inhibitors (lifirafenib, encorafenib, SB590885, GDC-0879, vemurafenib, dabrafenib, PLX7904, sorafenib, TAK-632, TAK-580, RAF709 and RAF265 were dissolved in DMSO (10 mM) and stored at −80°C. For *in vivo* use, lifirafenib and encorafenib were suspended in 0.5% carboxymethylcellulose with 0.5% Tween-80 in sterile water and dissolved via sonication. SB590885 was dissolved with stepwise addition of 2% N,N-dimethylacetamide and 2% Cremophor EL in acidified water (pH = 5.0).

#### Protein extraction

Protein lysates were obtained from cell pellets using an in-house lysis buffer (1 M Tris pH7.5, 5 M NaCl, and 0.5% (v/v) NP40, H2O, filtered) supplemented with protease and phosphatase inhibitors.

#### Immunoblotting

15 to 20 μg of protein/sample were mixed (4:1 v/v) with a mix of DL-Dithiothreitol (DTT) plus NuPAGE LDS Sample Buffer (1:10), incubated for 10 min at 95°C and then resolved on 10 to 12% acrylamide electrophoresis (running buffer: 0.25 M Tris-HCL pH 8.8, 1.9 M Glycine, 0.2% SDS) before being transferred to a PVDF membrane using a wet transfer method (transfer buffer: 200 nM Tris-HCL pH 7.5, 50 mM EDTA, 1 M NaCl). The membrane was washed with tris-buffered saline tween (TBST) before blocking with 5% (w/v) skimmed milk powder in TBST shaking for 1.5 h. The membrane was then incubated overnight with primary antibody at 4°C. After washing with TBST, the membrane was incubated for 1 h with an HRP-conjugated secondary antibody to detect the binding between the primary antibody and the protein. The chemoluminescent signal was detected by using homemade ECL and exposure in Advanced Molecular Vision Chemi Image Unit of the ChemoStar Imager. Antibodies used: polyclonal rabbit anti-human mitogen-activated protein (MAP) kinase [extra-cellular signal-regulated kinase (ERK) 1 & 2], monoclonal mouse anti-human MAP kinase, activated (diphosphorylated ERK-1 & 2) antibody) and monoclonal rabbit anti-human β-actin.

#### Proteomic/phospho-proteomic sample preparation

Cells were resuspended in 100 μL of ice-cold 8 M urea/50 Mm Tris HCL with phosphatase and protease inhibitors (Roche). Each sample was sonicated (Syclon Ultrasonic Homogenizer) for 2 × 9 s at a power setting of 15% to disrupt the cell pellet. The protein samples were normalised to 300 μg. Each sample was reduced by adding 8 mM dithiothreitol (dtt) and mixing (thermomixer 1200rpm, 30°C) for 60 min and carboxylated by adding 20 mM iodoacetamide and mixing (thermomixer 1200rpm, 30°C) for 30 min the dark. The solution was diluted with 50 mM Tris HCL to bring the urea concentration down to below 2 M. NB: (Urea must be below 2 M to prevent inhibition of trypsin). lyophilized trypsin (sequencing grade trypsin from Promega) was resuspended with 50 Mm Tris HCL at a concentration of 0.5 μg/μL and added to each solution. The samples were digested overnight with trypsin (1:100 enzyme to protein ratio) with gentle shaking (thermomixer 850rpm, 37°C). The digestion was terminated by adding formic acid to 1% final concentration and cleaned up using c18 (HyperSep SpinTip P-20, BioBasic C18, Thermo Scientific).

Phosphopeptide enrichment was carried out with TiO2 (Titansphere Phos-TiO Bulk 10 μm, (GL Sciences Inc, Tokyo, Japan) using an adapted method previously described (1). In summary, each sample was incubated with TiO2 beads for 30 min by rotation in 80% acetonitrile, 6% trifluoroacetic acid, 5 mM monopotassium phosphate, 20 mg/mL 2,5- dihydroxybenzoic acid, this step was carried out twice. The beads were washed 5 times in 80% acetonitrile/1% trifluoroacetic acid, before elution of the phosphopeptides with 50% acetonitrile, 7% ammonium hydroxide. The two eluents from each sample were then pooled and dried down.

#### Mass spectrometry

Samples were run on a Bruker timsTof Pro mass spectrometer connected to an Evosep One^51^ or a Bruker nanoElute chromatography system. The mass spectrometer was operated in positive ion mode with a capillary voltage of 1600 V, dry gas flow of 3 L/min and a dry temperature of 180 °C. All data was acquired with the instrument operating in trapped ion mobility spectrometry data dependent acquisition mode (TIMS DDA) mode. Trapped ions were selected for ms/ms using parallel accumulation serial fragmentation (PASEF). A scan range of (100–1700 m/z) was performed at a rate of 5 or 10 PASEF MS/MS frames to 1 MS scan with a cycle time of 1.03s or 1.89s^52^

#### Mass spectrometry data analysis

For analysis of MS output data, peptide mapping was initially performed with MaxQuant (release 2.0.1.0) using the Homo sapiens subset of the Uniprot Swissprot database with specific parameters for TIMS data dependent acquisition (TIMS-DDA). The MaxQuant output file was imported into the Perseus (version 1.6.15.0) environment for protein quantification. LFQ (Label Free quantitation) intensities were loaded as main columns. Reverse proteins and proteins only identified by site were filtered out from further analysis. LFQ values were then log2 transformed. The data frame was then split into smaller datasets by group of samples (e.g., condition v control) allowing filtering of proteins that were not identified in more than one replicate. Missing values were imputed based on the normal distribution with a width of 0.3 and a down shift of 1.8. These condition-specific (such as control condition and treated condition) data frames were then exported and used for further analysis.

For phosphoproteome analysis, label-free data was loaded into Perseus using “Intensity X_1,” “Intensity X_2,” “Intensity X_3” (i.e., data for the same sample and site for peptides that are mono-, di-, or tri-phosphorylated) as main columns. In addition to reverse proteins and proteins only identified by site, potential contaminants, and proteins with localization probability <0.75 were excluded from further analysis. The built-in function “Expand site table” was then used to rearrange the columns to permit analysis of differing levels of phosphorylation (i.e., single, double, triple-phosphorylated). Following log2 transformation, the position within the protein, known sites of phosphorylation, and linear motifs were all added to the table based on PhosphoSitePlus data (https://www.phosphosite.org). Similar to the whole proteome data analysis, the data frame was divided based on the condition, proteins filtered based on the number of replicates in which they appeared before imputing the missing values and exporting the data.^53^

Mass spectrometry proteomics data have been deposited to the ProteomeXchange Consortium via the PRIDE^54^ partner repository with the dataset identifier PXD063606.

Data can be accessed using the private token: tNSc6DiTCroL.

#### Kinase-substrate enrichment analysis (KSEA)

KSEA analysis (version 0.99.0) was performed in Jupyter Notebook coupled with R version 4.4.1. As previously described^55^ this approach allows inference of kinase activity based on the collective phosphorylation changes of their identified substrates, as determined from previously analyzed curated datasets. Files that were previously processed in Perseus and R (as described above) were modified to fit the KSEA pipeline requirements, as follows: “Protein” the Uniprot ID for the parent protein, “Gene” the HUGO gene name for the parent protein, “Peptide” the peptide sequence, “Residue.Both” all phospho-sites from that peptide, separated by semicolons (if applicable, these were formatted as the single amino acid abbreviation with the residue position (e.g., S102)), “p” the *p*-value of that peptide, and “FC” the fold change (not log-transformed). Arguments necessary to run the pipeline are as follow: ‘NetworKIN’ (TRUE or FALSE) to define if NetworKIN prediction should be included or not, ‘m.cutoff’ (numeric value from 1 to infinity) that indicates the minimum number of substrates to be included in the barplot output, and ‘p.cutoff’ (numeric value between 0 and 1) that indicates the *p*-value cut-off for inclusion of significant kinases in the bar plot.

#### Structure-based modeling of RAS/RAF/MEK/ERK signaling

To predict ERK phosphorylation dynamics in the venous blood, spleen, and bone marrow compartments, we developed and integrated a physiologically based pharmacokinetic (PBPK) model (see Data S1) with our structure-based MAPK signaling model.^28^

In the previous structure-based models,^16^^,^^23^^,^^28^^,^^41^ RAF inhibitor concentrations were fixed over time. Here, we dynamically updated inhibitor concentrations by the time-resolved drug concentration profiles predicted by the PBPK model for the 14 relevant compartments and specific RAFi (see Data S1 for details). This enabled the integrated model to capture the temporal dynamics of the drug exposure and to predict the inhibition of the RAS/RAF/MEK/ERK pathway in real time.

To match the experimental dosing regimen in mice, the model accounted for (i) SB590885 (Type I RAFi) administered intraperitoneally every 24 h, (ii) encorafenib (Type I½ RAFi) administered orally every 24 h, and (iii) lifirafenib (Type II RAFi) administered orally every 12 h, both as single drugs and in combinations.

The integrated model was built using the rule-based PySB framework.^56^ The SBML files of the model used in this study are available in Supplementary Information.

#### RAS mutation detection

Polymerase chain reaction (PCR) products for exon 2 of the *NRAS* gene was amplified from genomic DNA (gDNA) using Phusion High-Fidelity DNA Polymerase (New England BioLabs, Hitchin, UK) according to manufacturer’s instructions, with 35 cycles of amplification and an annealing temperature of 60.5°C. Primer sequences used were FWD: 5′-GCTCGCCAATTAACCCTGATTAC-3′, REV: 5′-TGGGTAAAGATGATCCGACAAGTGA-3’. Sanger sequencing was performed by Eurofins Genomics (Ebersburg, Germany).

#### Cell viability assays

For assessment of drug responses, cells were seeded in 96-well U-bottom plates (10,000 cells/well for cell lines and 50,000 cells/well for primary and PDX samples) prior to addition of inhibitors. Cell viability was assessed at 72 h using the Resazurin assay (made in house; 10*X* stock: resazurin 75 mg, methylene blue 12.5 mg, potassium hexacyanoferrate (III) 164.5 mg, potassium hexacyanoferrate 211 mg in 50 mL PBS). Cells were incubated with Resazurin solution (1:10 v/v) for 4 h and fluorescence measured at 560/590 nm using a SpectraMax M3 plate reader (Molecular Devices) SoftMaxPro 6.2.2.

Combination experiments were performed using both fixed-ratio and matrix (6 × 6) treatments. The Loewe Additivity method was used to determine combination effect, given the shared intracellular target of both agents. Loewe Additivity scores were calculated using the SynergyFinder web application (v3.0; https://synergyfinder.fimm.fi/).

#### Physiologically based pharmacokinetic model (PBPK)

We consider the following 16 compartments in the model: depot for oral administration, depot for intraperitoneal injection, lung, arterial, venous, adipose, muscle, liver, gut, spleen, heart, brain, kidney, skin, bone marrow, and the rest of the body. Total amounts of the drug (in milligrams) in each compartment are denoted as *D*_*PO*_, *D*_*IP*_, *A*_*lu*_, *A*_*ar*_, *A*_*ve*_, *A*_*ad*_, *A*_*mu*_, *A*_*li*_, *A*_*gu*_, *A*_*sp*_, *A*_*he*_, *A*_*br*_, *A*_*ki*_, *A*_*sk*_, *A*_*BM*_, *A*_*re*_. Concentration *C*_*i*_ of the drug in compartment *i* is calculated as *C*_*i*_ = *A*_*i*_/*V*_*i*_, where *V*_*i*_ is volume of this compartment. The blood flows through lungs, adipose, muscle, gut spleen, heart, brain, kidney, skin, bone marrow and rest of the body are denoted as, *Q*_*lu*_, *Q*_*ad*_, *Q*_*mu*_, *Q*_*gu*_, *Q*_*sp*_, *Q*_*he*_, *Q*_*br*_, *Q*_*ki*_, *Q*_*sk*_, *Q*_*BM*_, *Q*_*re*_, respectively. For the liver, the flow through hepatic artery is denoted as *Q*_*hepa*_, and hepatic venous flow is *Q*_*li*_ = *Q*_*hepa*_+*Q*_*gu*_+*Q*_*sp*_. Coefficient *BP* reflects blood to plasma ratio, coefficient *f*_*up*_ reflects fraction of the drug unbound in plasma, coefficients *K*_*p*_ reflect tissue to plasma partition coefficients for each compartment (Figure S6).

The drug from oral administration (PO) comes to the gut, the drug from intraperitoneal injection (IP) comes to venous blood. All RAF inhibitors are highly lipophilic small-molecule kinase inhibitors, a class of compounds that are predominantly cleared via hepatic metabolism and biliary/fecal excretion, with minimal renal contribution. Thus, we neglect renal clearance of drugs, and take into account only clearance in liver, denoted as *CL*. In such case, PBPK equations read as follows:

(Equation 1) $$\frac{dD_{PO}}{dt}=-K_{aPO}\cdot D_{PO}$$

(Equation 2) $$\frac{dD_{IP}}{dt}=-K_{aIP}\cdot D_{IP}$$

(Equation 3) $$\frac{dA_{lu}}{dt}=Q_{lu}\cdot \left(C_{ve}-C_{lu}\cdot \frac{BP}{K_{plu}}\right)$$

(Equation 4) $$\frac{dA_{ar}}{dt}=Q_{lu}\cdot C_{lu}\cdot \frac{BP}{K_{plu}}-\left(Q_{ad}+Q_{BM}+Q_{br}+Q_{he}+Q_{mu}+Q_{sk}+Q_{ki}+Q_{hepa}+Q_{gu}+Q_{sp}+Q_{re}\right)\cdot C_{ar}$$

(Equation 5) $$\frac{dA_{ve}}{dt}=K_{aIP}\cdot D_{IP}+Q_{ad}\cdot C_{ad}\frac{BP}{K_{pad}}+Q_{mu}\cdot C_{mu}\frac{BP}{K_{pmu}}+Q_{li}\cdot C_{li}\frac{BP}{K_{pli}}+Q_{he}\cdot C_{he}\frac{BP}{K_{phe}}+Q_{br}\cdot C_{br}\frac{BP}{K_{pbr}}+Q_{ki}\cdot C_{ki}\frac{BP}{K_{pki}}+Q_{sk}\cdot C_{sk}\frac{BP}{K_{psk}}+C_{BM}\frac{BP}{K_{pBM}}+Q_{re}\cdot C_{re}\frac{BP}{K_{pre}}-Q_{lu}\cdot C_{ve}$$

(Equation 6) $$\frac{dA_{ad}}{dt}=Q_{ad}\cdot \left(C_{ar}-C_{ad}\cdot \frac{BP}{K_{pad}}\right)$$

(Equation 7) $$\frac{dA_{mu}}{dt}=Q_{mu}\cdot \left(C_{ar}-C_{mu}\cdot \frac{BP}{K_{pmu}}\right)$$

(Equation 8) $$\frac{dA_{li}}{dt}=Q_{hepa}\cdot C_{ar}+Q_{gu}\cdot C_{gu}\frac{BP}{K_{pgu}}+Q_{sp}\cdot C_{sp}\frac{BP}{K_{psp}}-Q_{li}\cdot C_{li}\frac{BP}{K_{pli}}-C_{li}\cdot f_{up}\cdot CL$$

(Equation 9) $$\frac{dA_{gu}}{dt}=K_{aPO}\cdot D_{PO}+Q_{gu}\cdot \left(C_{ar}-C_{gu}\cdot \frac{BP}{K_{pgu}}\right)$$

(Equation 10) $$\frac{dA_{sp}}{dt}=Q_{sp}\cdot \left(C_{ar}-C_{sp}\cdot \frac{BP}{K_{psp}}\right)$$

(Equation 11) $$\frac{dA_{he}}{dt}=Q_{he}\cdot \left(C_{ar}-C_{he}\cdot \frac{BP}{K_{phe}}\right)$$

(Equation 12) $$\frac{dA_{br}}{dt}=Q_{br}\cdot \left(C_{ar}-C_{br}\cdot \frac{BP}{K_{pbr}}\right)$$

(Equation 13) $$\frac{dA_{ki}}{dt}=Q_{ki}\cdot \left(C_{ar}-C_{ki}\cdot \frac{BP}{K_{pki}}\right)$$

(Equation 14) $$\frac{dA_{sk}}{dt}=Q_{sk}\cdot \left(C_{ar}-C_{sk}\cdot \frac{BP}{K_{psk}}\right)$$

(Equation 15) $$\frac{dA_{BM}}{dt}=Q_{BM}\cdot \left(C_{ar}-C_{BM}\cdot \frac{BP}{K_{pBM}}\right)$$

(Equation 16) $$\frac{dA_{re}}{dt}=Q_{re}\cdot \left(C_{ar}-C_{re}\cdot \frac{BP}{K_{pre}}\right)$$

The table below shows compartment-specific parameters (Kp values were calculated using Rodgers & Rowland method^57^). Some studies suggest that the bone marrow functions as a “pharmacological sanctuary,” where drug penetration is limited due to protective mechanisms within the microenvironment.^58^ This sanctuary effect is attributed to factors such as the expression of drug-metabolizing enzymes like CYP3A4 by bone marrow stromal cells,^59^ which can inactivate therapeutic agents, and the presence of drug efflux transporters like P-glycoprotein and MRP1 that reduce intracellular drug accumulation.^60^ To phenomenologically reflect this reduced drug penetration in the bone marrow compartment, the Kp values were decreased by a factor of 1.25.

The volumes and blood flows were taken from the literature (Table S3).^10^^,^^61^

According to conservation laws the following equality must be satisfied:

(Equation 17) $$Q_{lu}=Q_{ad}+Q_{BM}+Q_{br}+Q_{he}+Q_{mu}+Q_{sk}+Q_{ki}+Q_{hepa}+Q_{gu}+Q_{sp}+Q_{re}$$

To estimate intrinsic hepatic clearance *CL*, which enters Equation 8, we used the well-stirred model.^62^ This model connects apparent hepatic clearance *CL*_*H*_, which is usually reported in literature, with intrinsic hepatic clearance *CL*

(Equation 18) $$CL_{H}=Q_{li}\cdot \left(\frac{CL\cdot f_{up}}{Q_{li}+CL\cdot f_{up}}\right)$$

Apparent hepatic clearance *CL*_*H*_ was rescaled from human data using allometric scaling *CL*_*H*_∝*W*^0.75^, where *W* is weight.^63^^,^^64^ These values can be adjusted based on obtained PK curves.

Using Equation 18, allometric scaling and publicly available data,^65^ we estimated other compound-specific parameters (Table S4).

*K*_*aPO*_ were estimated based on *T*_*max*_ times as *K*_*aPO*_ = 1.44/*T*_*max*_. We did not rescale *K*_*a*_ from human to mice using allometric scaling, since (i) absorption rates of other RAF inhibitors lie in the range of 0.5–1 L/h, not 5 L/h, and (ii) absorption rates are not well scaled with weight, unlike clearance rates. *K*_*aPO*_ for SB590885 was taken twice higher than *K*_*aPO*_ for encorafenib.

All unknown parameters for SB590885 are suggested to take from encorafenib. Initial values for *D*_*PO*_ and *D*_*IP*_ are taken from dosing regime.

#### Integration of the PBPK and structure-based MAPK signaling models

To predict ERK phosphorylation dynamics in the venous blood, spleen, and bone marrow compartments, we integrated a physiologically based pharmacokinetic (PBPK) model with our structure-based MAPK signaling model. Rather than seeking a point-by-point quantitative fit to specific experimental datasets, the primary objective of this integrated framework is to provide robust qualitative and mechanistic predictions exploring the interplay between compartment-specific pharmacokinetics and localized ERK signaling dynamics. This model is adapted from our previous work,^28^ with the PI3K-AKT module omitted for simplicity. The resulting model captures key intracellular processes, including ERBB receptor dimerization, recruitment of adaptor proteins to the plasma membrane, RAS activation via SOS1-mediated GDP-to-GTP exchange, RAF dimerization, ERK activation, and negative feedback loops from phosphorylated ERK to RAF, SOS1, and ERBB receptors. Allosteric effects induced by conformation-specific RAF inhibitors (RAFi) are quantitatively represented using thermodynamic factors.^23^

In the previous structure-based model,^28^ RAFi concentrations were fixed over time. Here, we dynamically updated RAFi concentrations by directly linking the RAFi concentration variables to the time-resolved drug concentration profiles predicted by the PBPK model in the relevant compartments. This allowed the integrated model to capture the temporal dynamics of the drug exposure.

To match the experimental dosing regimen in mice, SB590885 (Type I RAFi) was administered intraperitoneally every 24 h, encorafenib (Type 1½ RAFi) was administered orally every 24 h, and lifirafenib (Type II RAFi) was administered orally every 12 h.

The integrated model was built using the rule-based PySB framework.^56^ The SBML files of the model used in this study are available in Supplementary Information.

#### Sensitivity analysis

To assess the robustness of the model outcomes, and to address the inherent quantitative uncertainty of model predictions, we performed sensitivity analysis, which examines how perturbations to model parameters affect the cumulative ppERK responses. The sensitivity coefficients are calculated as the percent change in the ppERK response caused by 1% change of a model parameter, and are defined by

(Equation 19) $$\begin{array}{c} s_{i}=\frac{d\;\ln\;M}{d\;\ln\;p_{i}}\text{,} \end{array}$$

where *M* is the model outcome, i.e., AUC of ppERK responses, and *p*_*i*_ is the i-th model parameter. Sensitivity coefficients were calculated using finite difference approximations with 1% changes in the reaction rates.

#### Assessment of *in vivo* efficacy

The AML005a PDX was obtained from the Baylor College of Medicine Patient-Derived Xenograft Core. AML005a cells (1 × 10^6^) were inoculated intravenously (i.v.) into unconditioned NSGS mice (6–8 weeks of age). Mice were bled (via tail vein) weekly to determine the percentage of human CD45^+^ (hCD45^+^) cells vs. mouse CD45^+^ (mCD45^+^) cells in peripheral blood (PB) using flow cytometry. When the median engraftment reached ≥1% hCD45^+^ cells, mice were assigned to treatment groups (*n* = 8). Mice were treated with either lifirafenib (10 mg/kg PO twice daily), encorafenib (20 mg/kg PO once daily), SB590885 (25 mg/kg IP once daily), combined lifirafenib + encorafenib, combined lifirafenib + SB590885 or vehicle control. Representative mice were euthanized at day 10, with BM and spleen harvested to assess leukemia burden and on-target efficacy of treatments. Mice were maintained in the experiment until they reached event, deemed 25% hCD45^+^ cells in PB.

Event-free survival (EFS) for individual mice was calculated as the number of days from treatment initiation until event, with the time of event calculated by interpolating between bleeds directly pre- and post-event, assuming log-linear growth. Leukemia growth delay (LGD) was measured as treatment minus control (T-C) in days. To evaluate interactions between drugs *in vivo*, therapeutic enhancement was considered if the EFS of mice treated with the combination treatment was significantly greater (*p* < 0.05) than those induced by both single agents.

### Quantification and statistical analysis

Statistical analyses were performed using GraphPad Prism (v8.0.2).

For PDX studies, event-free survival (EFS) for individual mice was calculated as the number of days from treatment initiation until event, with the time of event calculated by interpolating between bleeds directly pre- and post-event, assuming log-linear growth. Leukemia growth delay (LGD) was measured as treatment minus control (T-C) in days using median EFS values of each treatment group. The number of evaluable mice per treatment cohort is provided in the Results section. Statistical comparison of Kaplan-Meier survival curves was performed using the Log rank (Mantel-Cox) test. To evaluate interactions between drugs *in vivo*, therapeutic enhancement was considered if the EFS of mice treated with the combination treatment was significantly greater (*p* < 0.05) than those induced by both single agents, as determined by unpaired two-tailed t tests. To compare site-specific leukemia burden at Day 10 during treatment, unpaired two-tailed t tests were used and significance level defined by asterisks (∗*p* < 0.05, ∗∗*p* < 0.01).

Quantification of mass spectrometry data was performed using MaxQuant (release 2.0.1.0), Perseus (v1.6.15.0). Subsequent KSEA analysis (v0.99.0) was performed in Jupyter Notebook coupled with R (v4.4.1).

All statistical details, including *n*, replicates and test type are provided in the relevant figure legends.

## Supplemental information

Document S1. Figures S1–S6 and Tables S1–S4

Data S1. Integrated modeling files for structure-based modeling and PBPK modeling, relating to Figures 1C, 4A and 5A
