# Supplementary material for: A structure-based modeling approach identifies effective drug combinations for RAS-mutant acute myeloid leukemia
Source: iScience. 2026 Jul 20;29(8):116875. doi: 10.1016/j.isci.2026.116875 (PMC13393809; doi:10.1016/j.isci.2026.116875)
Supplement: Document S1. Figures S1–S6 and Tables S1–S4 [file mmc1.pdf]

iScience, Volume *29*

Supplemental information

A structure-based modeling approach identifies effective drug combinations for RAS-mutant acute myeloid leukemia

Luke Jones, Oleksii Rukhlenko, Tânia Dias, Hiroaki Imoto, Ciardha Carmody, Kieran Wynne, Boris N. Kholodenko, and Jonathan Bond

SUPPLEMENTAL DATA

Contents:

- Supplemental Figures S1-S6
- Supplemental Tables S1-4

Supplemental Figures

Figure S1

A

Cell line	RAS mutation	Additional alterations	Age (Yrs)	Sex
OCI-AML3	*NRAS*^{Q61L}	*DNMT3A*^{R882C}<br>NPM1c+	57	M
HL-60	*NRAS*^{Q61L}	*TP53*^{null}<br>*CDKN2A* Hom c.238C>T	36	F
THP-1	*NRAS*^{G12N}	*KMT2A::MLLT3*<br>*CSNK2A1::DDX39B*<br>*TP53* Het c.520_545del26	1	M
NB4	*KRAS*^{A18D}	*PML::RARA*<br>*TP53* Hom c.743G>A	23	F
ML-2	*KRAS*^{A146T}	*KMT2A::AFDN*	26	M

B

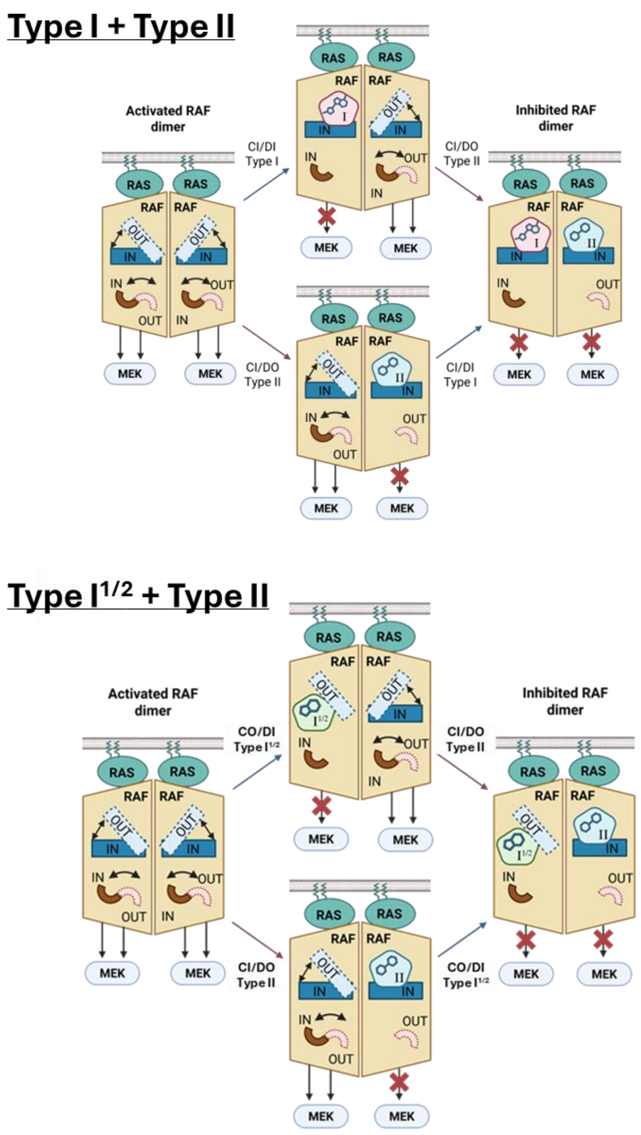

Figure S1. Cell line characteristics and diagrammatic representation of conformation-specific RAF inhibitor binding to asymmetric RAF dimers. (A) Characteristics of the AML cell lines used in this study. **(B)** Cartoon depicting the binding preferences and effects on downstream signaling upon treatment with Type I, Type II and Type I + Type II RAF inhibitors. Schema adapted from Rukhlenko et al., 2018.

Figure S2

A

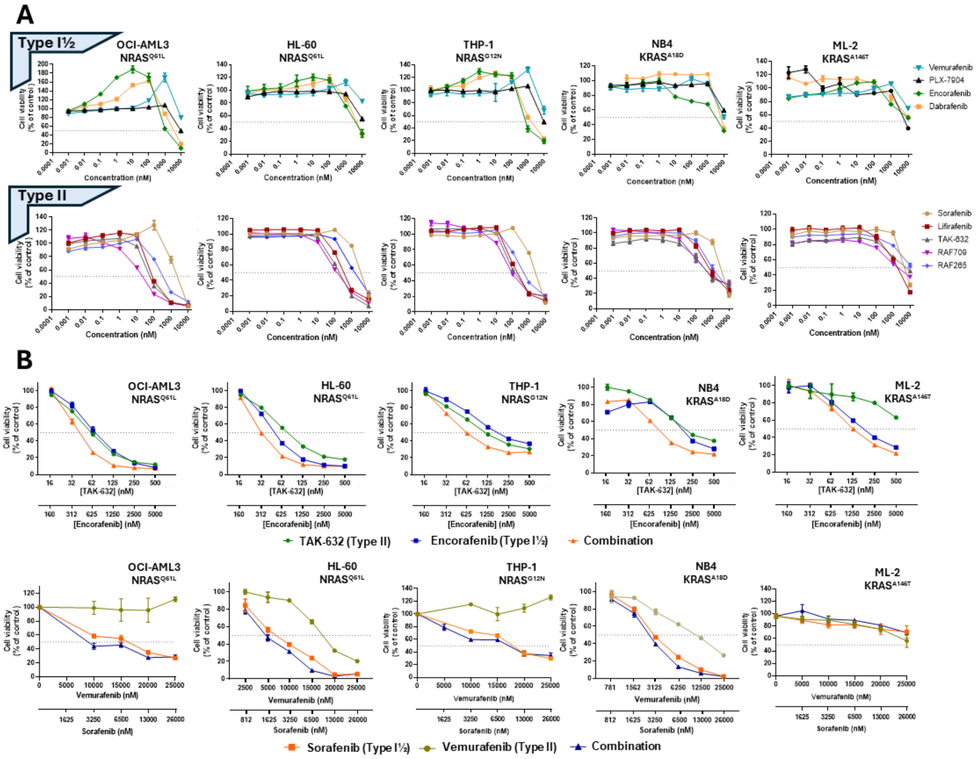

C

Cell line	Loewe additivity scores	
	TAK-632 + encorafenib	Vemurafenib + sorafenib
OCI-AML3	-13.54	-7.73
HL60	-10.21	-9.81
THP-1	-8.7	-10.4
NB4	-7.96	-14.74
ML-2	-5.06	-9.17

D

Sample	RAS mutation	Other alterations	Sex	Age @ Dx (Yrs)
7638	NRAS^{G12V}	KMT2A::KIAA1524	F	0.4
MC46	NRAS^{G12D}	Inv(16)(p13q22)	M	16

E

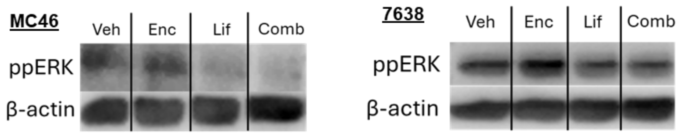

F

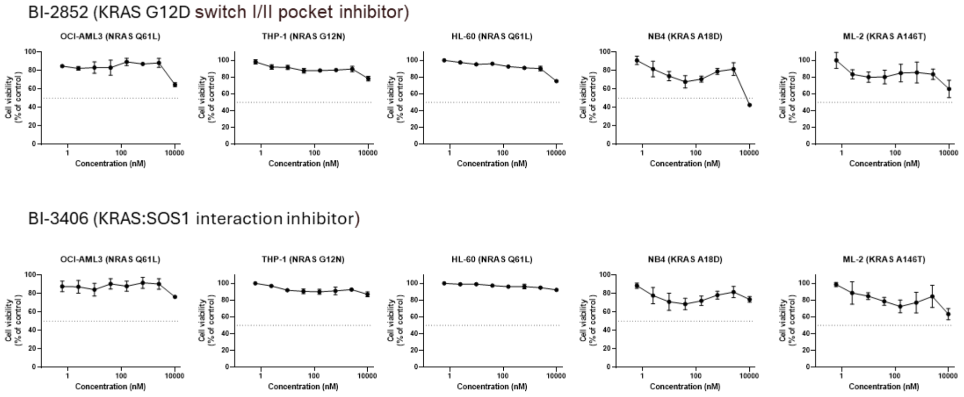

Figure S2. Additional in vitro and ex vivo dose responses, including single agent RAF inhibitor responses, alternative RAF inhibitor combinations, patient sample characteristics and responses and efficacy of RAS inhibitors. (A) Single-agent dose-response curves for all Type I½ and Type II RAF inhibitors tested against five *RAS*-mutant cell lines. **(B)** Cell viability in response to TAK-632 (Type II) + encorafenib (Type I½) or sorafenib (Type I½) + vemurafenib (Type II) compared with each single agent against *RAS*-mutant cell lines. **(C)** Loewe Additivity scores for combinations in Fig. S2B. **(D)** Characteristics of primary AML samples used for *ex vivo* drug treatments. **(E)** Immunoblots for MC46 (top) and 7638 (bottom) measuring ppERK levels relative to vehicle, single agent or combination treatment (24h). **(F)** Single-agent dose response for RAS-specific inhibitors. For cell viability experiments, cell viability was determined using AlamarBlue at 72 hours post-treatment. Data points represent mean + SEM from 3 replicate experiments.

Figure S3

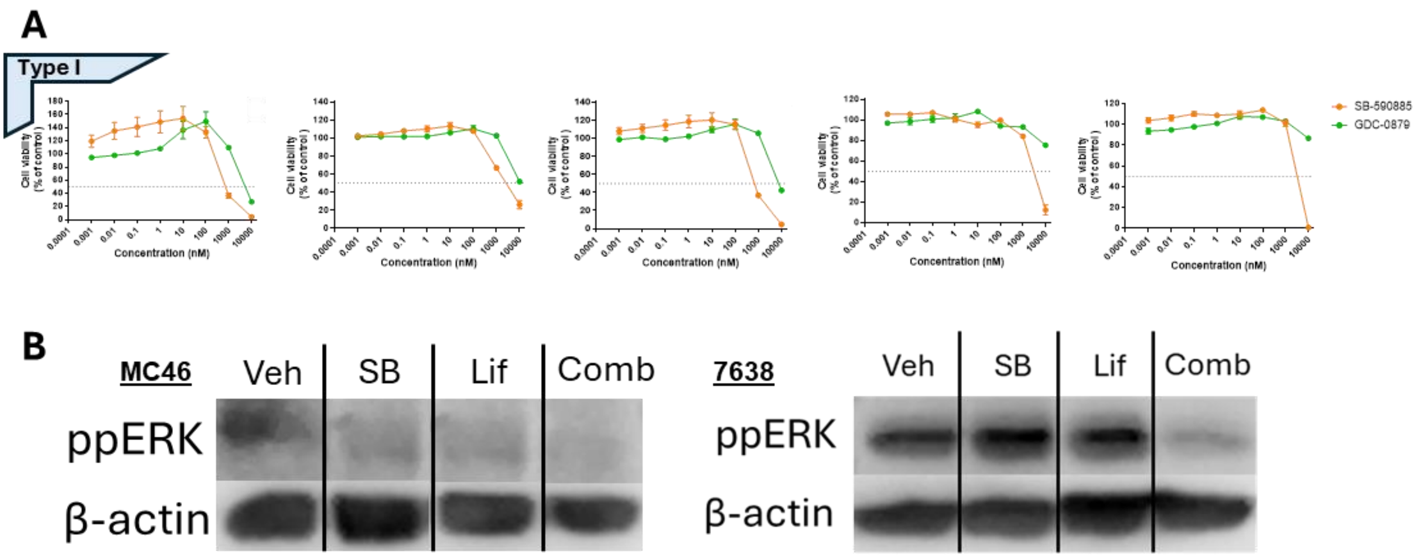

Figure S3. Additional in vitro and ex vivo RAF inhibitor response data, including single agent RAF inhibitor efficacy and patient sample responses to combination treatment. (A) Single-agent dose-response curves for all Type I RAF inhibitors tested against five *RAS*-mutant cell lines. **(B)** Immunoblots for MC46 (left) and 7638 (right) measuring ppERK levels relative to vehicle, single agent or combination treatment (24h).

Figure S4

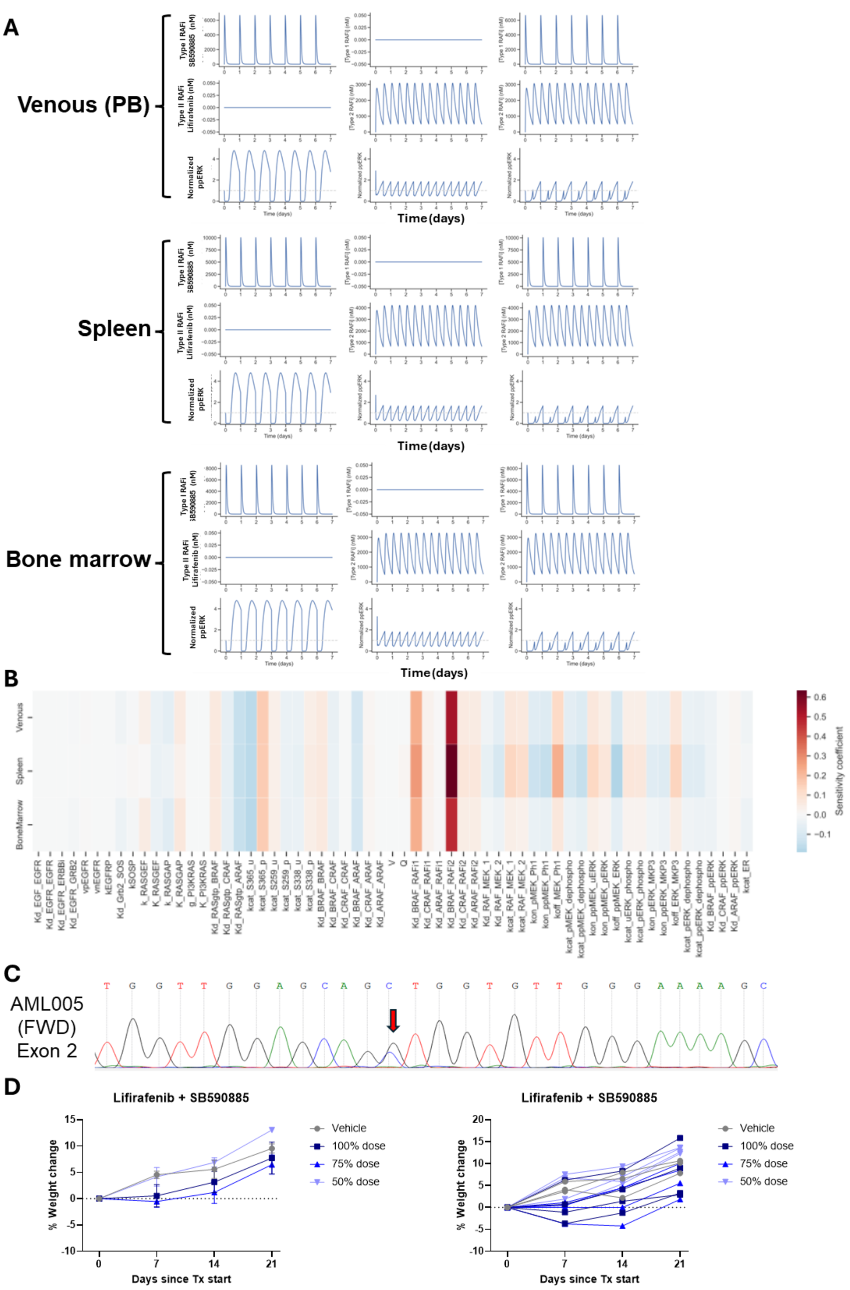

E

Treatment group	Median event-free survival (**EFS**) (Days)	Median leukemia growth delay (**LGD**) (T-C; Days)
Vehicle	5.08	-
Lifirafenib	8.50	4.39
SB590885	13.79	8.71
Combination	13.99	8.91

Figure S4. Supporting data for in vivo experiments using Type I and Type II RAF inhibitors, including predicted drug concentrations and pathway inhibition, validation of RAS mutation, tolerability and event-free survival / leukemia growth delay. (A) Area under the curve (AUC) for prediction of ppERK levels over time during treatment in venous blood (PB), spleen and bone marrow. **(B)** Sensitivity of cumulative ERK phosphorylation responses to perturbations in the venous, spleen, and bone marrow models. Simulations were done in the presence of Type I½ RAFi Encorafenib and Type II RAFi Lifirafenib. Colors represent sensitivity coefficients for the parameters listed on the x-axis. Parameter prefixes denote: Kd, equilibrium dissociation constant; kon, binding rate constant; koff, dissociation rate constant; kcat (and k,), catalytic rate constant. The parameters V and Q denote the volume and spleen blood flow at each compartment, respectively. **(C)** Sanger sequencing showing point mutation in Exon 2 of *NRAS* in AML005. **(D)** Tolerability data for lifirafenib + SB590885 treatment as determined by weight loss over time. **(E)** Leukemia growth delay (LGD) values calculated for AML005 treated with combination or single agents.

Figure S5

A

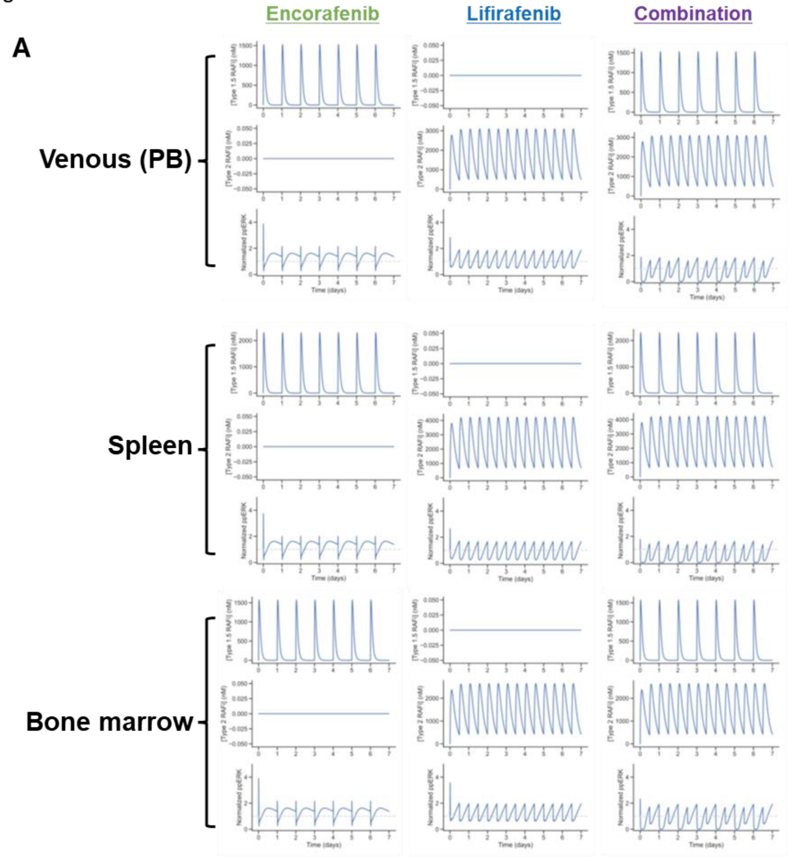

B

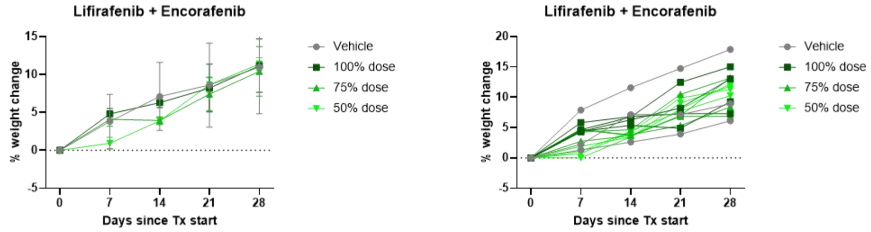

C

Treatment group	Median event-free survival (**EFS**) (Days)	Median leukemia growth delay (**LGD**) (T-C; Days)
Vehicle	5.08	-
Lifirafenib	8.50	4.39
Encorafenib	4.83	-0.25
Combination	11.91	6.83

Figure S5. Supporting data for in vivo experiments using Type I1/2 and Type II RAF inhibitors, including predicted drug concentrations and pathway inhibition, tolerability and event-free survival / leukemia growth delay. **(A)** Area under the curve (AUC) for prediction of ppERK levels over time during treatment in venous blood (PB), spleen and bone marrow. **(B)** Tolerability data for lifirafenib + encorafenib treatment as determined by weight loss over time. **(D)** Leukemia growth delay (LGD) values calculated for AML005 treated with combination or single agents.

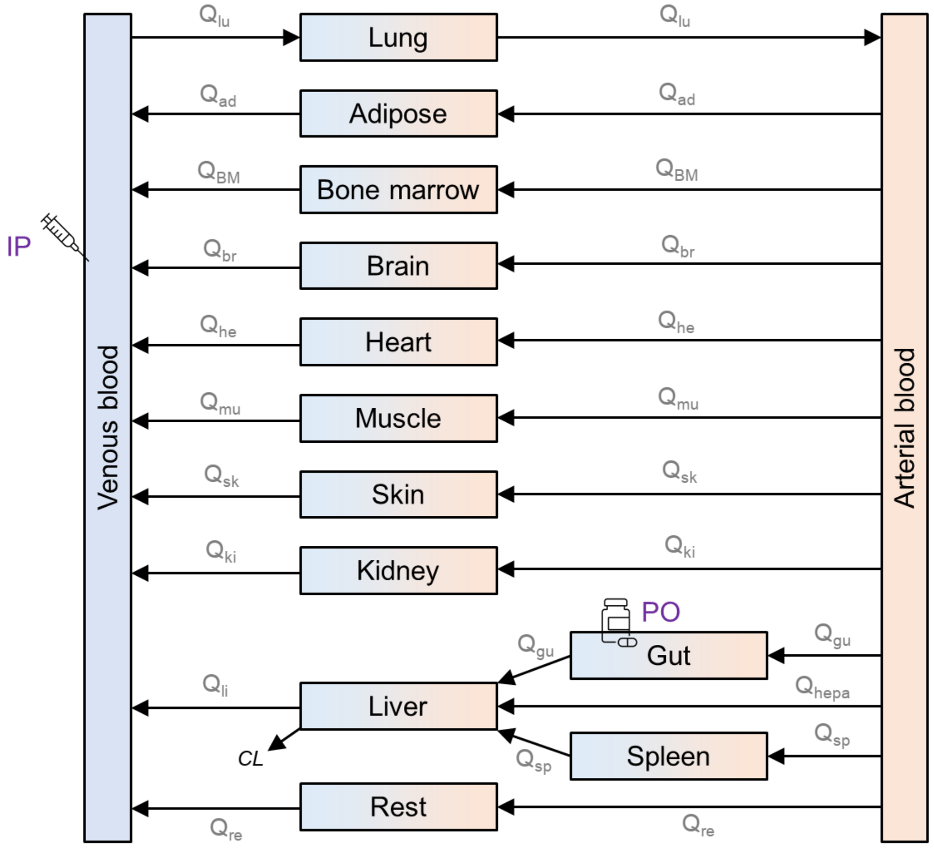

Figure S6. Schematic of blood-flow parameters through tissue. Calculated blood-flow in different anatomical compartments. Relevant sites of drug administration (IP, PO) are shown.

Supplemental Tables

Cell line	HSA Synergy Score	BLISS Additivity Score
**OCI-AML3**	10.97	3.10
**HL60**	17.42	5.77
**THP-1**	19.96	6.44
**NB4**	15.33	2.07
**ML-2**	10.98	2.94

Supplementary Table S1. Alternative synergy metrics applied to *in vitro* data for the lifirafenib + encorafenib combination.

Cell line	HSA Synergy Score	BLISS Additivity Score
**OCI-AML3**	6.38	7.43
**HL60**	15.52	7.39
**THP-1**	11.43	4.64
**NB4**	4.32	-6.97
**ML-2**	-7.64	-5.05

Supplementary Table S2. Alternative synergy metrics applied to *in vitro* data for the lifirafenib + SB590885 combination.

Compartment	Volume, L	Blood flow, L/h	Kp, Lifirafenib	Kp, Encorafenib
Lung	2.06E-04	1.2000	1.7710	1.7715
Arterial	7.42E-04	1.2000	1.0000	1.0000
Venous	1.47E-03	1.2000	1.0000	1.0000
Adipose	2.49E-03	0.0134	6.8305	4.4789
Muscle	1.20E-02	0.1604	0.7998	0.8541
Liver (Q_{hepa} for flow)	2.17E-03	0.0745	1.3660	1.3904
Gut	1.27E-03	0.2642	2.6753	2.6352
Spleen	1.31E-04	0.0159	0.7682	0.8202
Heart	1.42E-04	0.0494	1.2038	1.2361
Brain	5.42E-04	0.0803	2.4274	2.4098
Kidney	5.07E-04	0.2253	1.2931	1.3201
Skin	4.58E-03	0.1067	3.9718	3.8513
Bone Marrow	1.77E-03	0.1523	0.4800	0.5600
Rest	1.98E-03	0.0577	1.0000	1.0000
Total	3.00E-02	1.2000		

Supplementary Table S3. Volumes and blood flow values (taken from literature) for discrete anatomical sites, and the associated Kp values calculated for lifirafenib and encorafenib.

Parameter	Value for Encorafenib	Value for Lifirafenib	Value for SB590885
Clearance, CL, L/h	0.9312	3.1093	Same as Encorafenib
Blood-to-plasma, BP	0.5800	0.6000	Same as Encorafenib
K_{aPO}, 1/h	0.7200	0.4800	-
K_{aIP}, 1/h	-	-	1.4400
f_{up}	0.1400	0.0050	Same as Encorafenib

Supplementary Table S4. Calculated compound-specific parameters determined for our Physiologically Based Pharmacokinetic Model (PBPK).
